# CmVNI2-*CmMYB3* module regulates flavonol biosynthesis in response to low temperature in chrysanthemum flower

**DOI:** 10.1186/s43897-025-00175-x

**Published:** 2025-10-09

**Authors:** Tianhua Jiang, Yahui Sun, Yao Wang, Jiayi Luo, Lei Liu, Hanqin Chen, Yiwei Xue, Lili Wang, Kang Gao, Dongliang Chen, Chao Ma, Conglin Huang, Chang Luo

**Affiliations:** 1 Institute of Grassland, Flowers and Ecology, Beijing Academy of Agriculture and Forestry Sciences, Beijing, 100097 China; 2 Beijing Key Laboratory of Development and Quality Control of Ornamental Crops, Department of Ornamental Horticulture, College of Horticulture, China Agricultural University, Beijing, 100193 China; 3 College of Landscape Architecture, Beijing University of Agriculture, Beijing, 102206 China; 4 College of International Education, Beijing University of Chemical Technology, Beijing, 100029 China

**Keywords:** *Chrysanthemum morifolium*, CmVNI2, CmMYB3, Flavonol, Low temperature

## Abstract

Flavonols represent a large subgroup of flavonoids and function as the principal bioactive compounds in tea and medicinal chrysanthemum (*Chrysanthemum morifolium*) flowers. Low temperature is one of the most significant environmental factors influencing flavonol accumulation. Nevertheless, the regulatory mechanisms governing flavonol biosynthesis in response to low temperature remain predominantly uncharacterized. In this study, we observed decreased flavonol accumulation in chrysanthemum flowers under low-temperature conditions, correlating with reduced expression of the NAC transcription factor *VND-INTERACTING2* (*VNI2*). The suppression of CmVNI2 resulted in diminished flavonol content. DNA affinity purification sequencing and RNA sequencing analyses demonstrated that CmVNI2 directly regulates the expression of *CmF3H* and *CmMYB3*, two genes essential for flavonol biosynthesis. In addition, transient overexpression of *CmMYB3* in *CmVNI2* RNA interference plants restored flavonol accumulation. The study establishes that the CmVNI2–*CmMYB3* module plays a crucial role in regulating flavonol biosynthesis in chrysanthemum flowers under low-temperature stress and identifies potential target genes for enhancing the bioactive properties of chrysanthemum as a tea or medicinal herb.

**Supplementary Information:**

The online version contains supplementary material available at 10.1186/s43897-025-00175-x.

## Core

The NAC transcription factor CmVNI2 regulates flavonol biosynthesis through directly controlling the expression of *CmF3H* and *CmMYB3* in response to low temperature. Overexpression of *CmMYB3* in *CmVNI2* RNAi plants led to a restoration of flavonol accumulation. Our findings indicate that the CmVNI2-*CmMYB3* module plays a crucial role in regulating flavonol biosynthesis in chrysanthemum flowers under low-temperature stress.

## Gene & accession numbers

Gene sequence information was obtained from the chrysanthemum database (http://210.22.121.250:8880/asteraceae/homePage). The accession numbers of the genes used in this study are listed in Fig. 4c, Fig. S1, and Fig. S2.

## Introduction

Flavonoids, a diverse group of polyphenols, are essential metabolites that serve crucial roles in plant interactions with both biotic and abiotic environments (Dong and Lin 2021; He et al. 2023). They are classified into seven subgroups: flavones, flavonols, isoflavones, anthocyanidins, flavanones, flavanols, and chalcones, distinguished by their oxidation states and structural skeletons (Shen et al. 2022). Research on low-temperature stress has predominantly examined vegetative organs, demonstrating that cold stress induces the expression of genes in the flavonoid biosynthesis pathway, including chalcone synthase (*CHS*), chalcone isomerase (*CHI*), flavonol synthase (*FLS*), and dihydroflavonol 4-reductase (*DFR*), thereby enhancing flavonoid accumulation (He et al. 2023). However, flavonoid accumulation in reproductive organs demonstrates a contrasting response to low temperatures; for instance, anthocyanin accumulation in strawberry fruits is inhibited under these conditions (Mao et al. 2022). The mechanisms governing flavonoid accumulation in response to low temperatures remain largely unexplored.

Flavonols constitute one of the most widespread and abundant classes of flavonoids, primarily comprising kaempferol, quercetin, myricetin, and their derivatives. The flavonol biosynthesis pathway is a branch of the flavonoid biosynthesis pathway, which begins with *p*-coumaroyl-CoA and diverges into two main pathways: flavonoid and lignin biosynthesis pathways. The transformation of* p*-coumaroyl-CoA is catalyzed by CHS and hydroxycinnamoyl-CoA shikimate/quinate hydroxycinnamoyl transferase (HCT), key enzymes that direct metabolic flux toward flavonoid and lignin metabolism, respectively. In the flavonoid pathway, CHS catalyzes naringenin chalcone formation from *p*-coumaroyl-CoA. Subsequently, CHI converts naringenin chalcone to naringenin, a common precursor for other flavonoid classes. Flavanone 3-hydroxylase (F3H) competes with flavone synthase (FNSII) to transform naringenin into the flavonol precursor dihydrokaempferol, which FLS then converts to flavonols (Martens and Mithöfer 2005).

Flavonol biosynthesis is primarily regulated by the R2R3-MYB subgroup seven (SG7 MYB) transcription factors (TFs) (Stracke et al. 2007; Fernandez-Moreno et al. 2016; Huang et al. 2016). SG7 MYB family members regulate flavonol biosynthesis by directly binding to flavonol biosynthesis gene promoters to activate their expression (Mehrtens et al. 2005; Stracke et al. 2007). In *Arabidopsis*, the SG7 MYB factors MYB12, MYB11, and MYB111 specifically control the expression of *CHS*, *CHI*, *F3H*, and *FLS1* to regulate flavonol accumulation (Stracke et al. 2010; Xu et al. 2015). In chrysanthemum (*Chrysanthemum morifolium*), the SG7 MYB member *CmMYB3* functions as a transcriptional activator in flavonol biosynthesis regulation (Yang et al. 2023). In addition, SG19 MYB proteins participate in regulating flavonol biosynthesis in *Freesia hybrida*, *Arabidopsis*, and *Vitis vinifera* (Battat et al. 2019; Shan et al. 2020; Zhang et al. 2023). In *F. hybrida*, SG19 MYB proteins, including MYB21 and MYB24, regulate flavonols by modulating *FLS* expression (Shan et al. 2020). In *V. vinifera*, the SG19 MYB protein MYB24 coordinates terpene and flavonol metabolism (Zhang et al. 2023). However, the upstream regulators of MYB proteins involved in flavonol biosynthesis require further investigation.

The NAC (no apical meristem–*Arabidopsis* transcription activation factor–cup-shaped cotyledon) family represents one of the largest plant-specific TF families. These TFs play essential roles in various developmental processes, including cell proliferation, flowering time, secondary cell wall (SCW) biosynthesis, and responses to biotic and abiotic stresses (Puranik et al. 2012; Diao et al. 2020; Liu et al. 2022). For example, the *Arabidopsis* NAC domain transcriptional activator VASCULAR-RELATED NAC-DOMAIN7 (VND7) functions as a master regulator of xylem vessel element differentiation, while *MYB83* and *MYB46* are direct targets of VND7 and regulate genes involved in the biosynthesis of SCW and lignin (Yamaguchi et al. 2010; Nakano et al. 2015). Furthermore, the Arabidopsis NAC domain TF VND-INTERACTING2 (VNI2) acts as a transcriptional repressor that regulates xylem cell specification through its interaction with VND7 proteins (Yamaguchi et al. 2010). NAC TFs also participate in flavonoid biosynthesis. In Arabidopsis, ANAC078 promotes flavonoid biosynthesis by inducing the expression of *PAP1*, *GL3*, and *EGL3* under high-light conditions (Morishita et al. 2009). In blood-flesh peach, the BL-PpNAC1 heterodimer enhances anthocyanin pigmentation by activating *PpMYB10.1* transcription (Zhou et al. 2015). However, the role of NAC in regulating flavonoid biosynthesis under low-temperature conditions remains largely unexplored.

Chrysanthemum ranks among the most important ornamental plants worldwide. The optimum temperature for chrysanthemum growth ranges from 18 °C to 25 °C, while temperatures below 10 °C delay flowering or affect flower morphology (Bai et al. 2022; Hu et al. 2025). Chrysanthemum flowers have been traditionally used as tea or medicine in China for centuries. Flavone and flavonol constitute the two main classes of compounds in chrysanthemum, contributing to both flower coloration and various health-beneficial bioactivities (Hao et al. 2022). Flavones predominantly accumulate in chrysanthemum ray florets, while flavonols are mainly present in disc florets (Luo et al. 2022). This study identified an NAC TF, *CmVNI2*, whose expression levels correlate with flavonol content and low-temperature responses in chrysanthemum flowers. Functional analyses demonstrated that CmVNI2 plays a crucial role in regulating flavonol accumulation in response to low temperature.

## Results

### Flavonol accumulation and *CmVNI2* expression are reduced in chrysanthemum flowers in response to low temperature

**Fig. 1:**
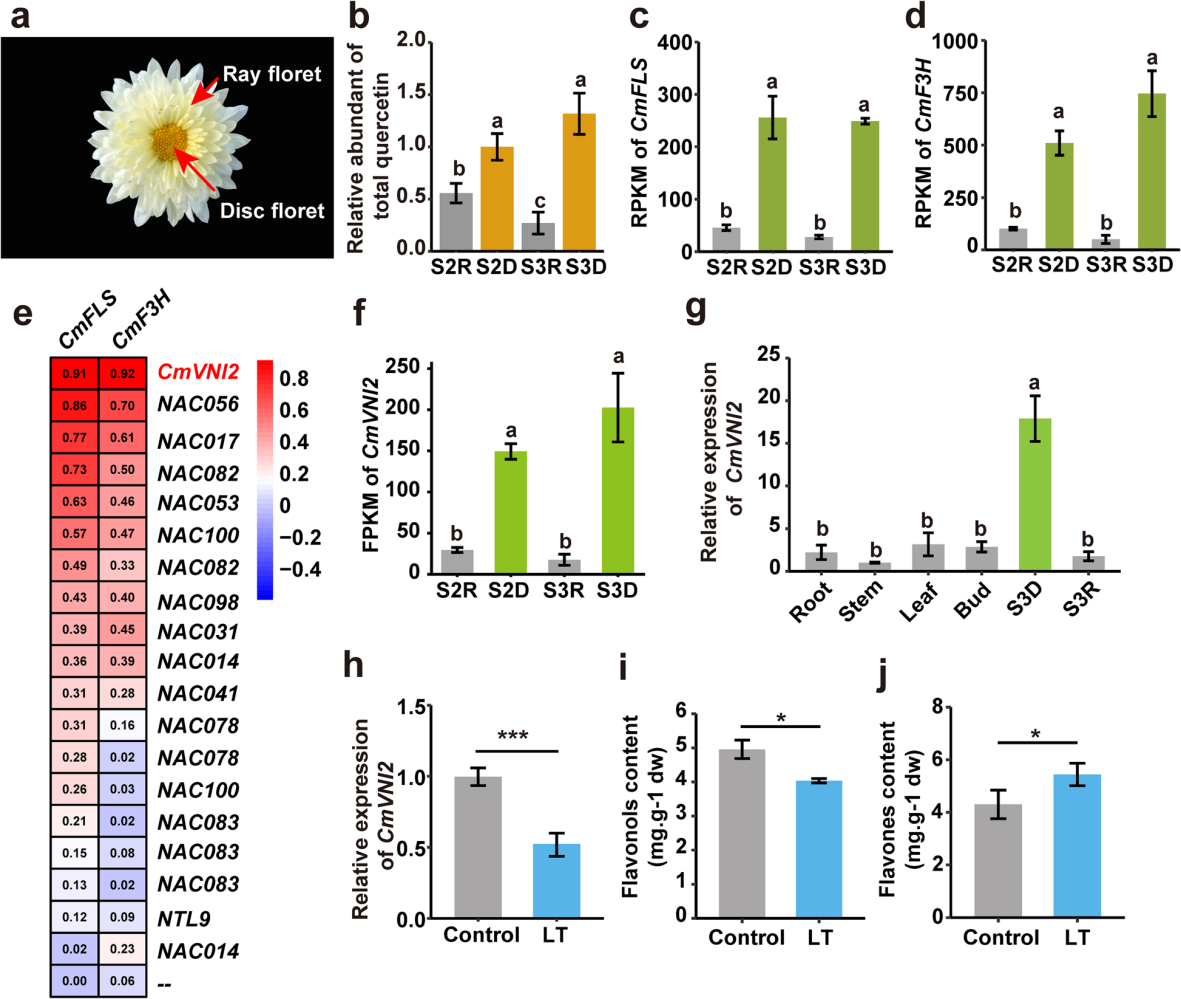
Accumulation of flavonols and expression of *CmVNI2* in chrysanthemum florets. **a** Morphological characteristics of the chrysanthemum capitulum. **b** Flavonol accumulation in ray and disc florets. S2D, disc floret at opening stage 2; S2R, ray floret at opening stage 2; S3D, disc floret at opening stage 3; S3R, ray floret at opening stage 3. Gene expression levels of *CmFLS* (**c**) and *CmF3H* (**d**) in ray and disc florets. **e** Heatmap of the co-expression between NAC TFs and *CmFLS* as well as *CmF3H*. Red indicates high correlation, while blue indicates low correlation. Numbers represent correlation coefficients. **f** Gene expression levels of *CmVNI2* in ray and disc florets. **g** qRT-PCR analysis of *CmVNI2* expression across different chrysanthemum tissues. **h** Effects of low temperature on *CmVNI2* gene expression. Control, at 22 °C; LT, after 7-day low-temperature exposure at 10 °C. **i** Effects of low temperature on flavonol (quercetin and kaempferol) accumulation. dw, dry weight. **j** Effects of low temperature on flavone (apigenin and luteolin) accumulation. Values are means ± SDs. Standard error was calculated from three biological replicates. Significantly different values (*P* < 0.05) are indicated with lowercase letters. Values were calculated by one-way analysis of variance (ANOVA) followed by Tukey’s honestly significant difference (HSD) test. Asterisks indicate significant differences between the control and low-temperature treatments (Student’s *t*-test; *P < 0.05; **P < 0.01; ***P < 0.001)

The chrysanthemum capitulum consists of numerous individual ray and disc florets (Fig. 1a). Previous metabolite analysis of *C. morifolium* ray and disc florets revealed significant accumulation of quercetin and its derivatives in disc florets (Fig. 1b). Similarly, the flavonoid biosynthesis genes, *CmFLS* and *CmF3H*, exhibited significantly higher expression in disc florets compared with ray florets (Fig. 1c, d). To identify the flavonol biosynthesis regulator in chrysanthemum, we performed a co-expression analysis between TFs and flavonoid biosynthesis genes using RNA sequencing (RNA-seq) data from ten flower samples (Supplementary Dataset S1). Based on correlation coefficient rankings, CmVNI2 was selected as a potential regulator for further investigation (Fig. 1e). Quantitative real-time PCR (qRT-PCR) analysis demonstrated that CmVNI2 expression levels were higher in disc florets compared with other organs, including roots, stems, leaves, and ray florets (Fig. 1f, g). CmVNI2 encodes a protein of 255 amino acids, and multiple sequence alignment revealed that this protein contains a typical NAC domain comprising five conserved amino acid motifs at its N-terminus (Fig. S1) (Olsen et al. 2005). Phylogenetic analysis indicated that CmVNI2 is closely related with AtVNI2 and belongs to the NAC protein subgroup involved in growth and SCW development (Fig. S2). To examine the effects of low-temperature conditions on *CmVNI2* expression and flavonoid accumulation in chrysanthemum flowers, we measured *CmVNI2* expression levels using qRT-PCR in flowers maintained at 22 °C (control) and after 7-day exposure at 10 °C. *CmVNI2* expression significantly decreased after 7-day low-temperature exposure (Fig. 1h). In addition, flavonol contents, including quercetin and kaempferol, showed significant reduction after 7-day low-temperature treatment (Fig. 1i). Conversely, flavone contents, comprising apigenin and luteolin, increased significantly after 7-day low-temperature treatment (Fig. 1j).

### *CmVNI2* regulates flavonol contents in chrysanthemum flowers in response to low temperature

**Fig. 2:**
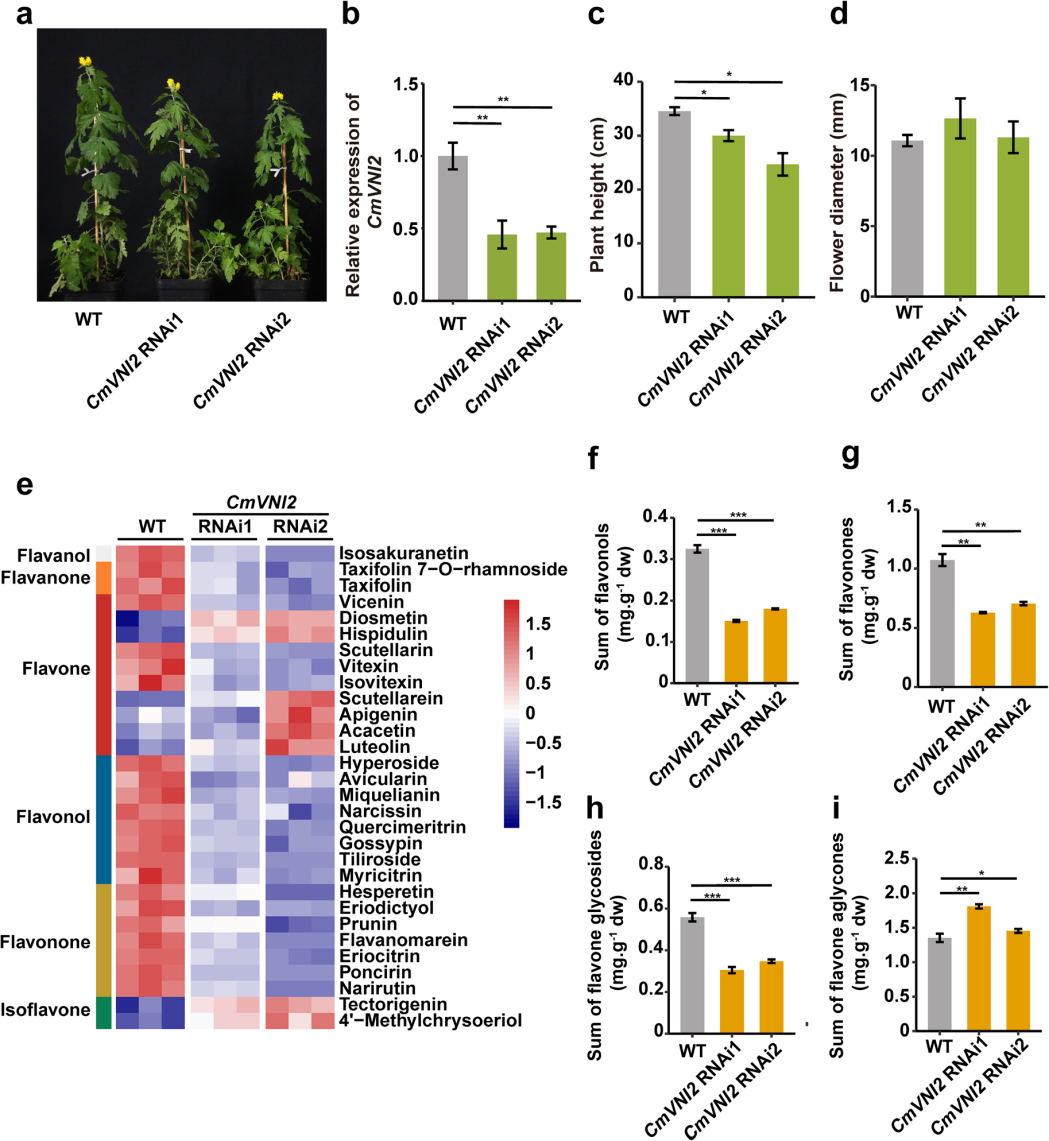
Flavonoid contents in flowers of wild type (WT) and *CmVNI2* RNAi lines. **a** Phenotypes of WT and *CmVNI2* RNAi lines. **b** qRT-PCR analysis of *CmVNI2* transcript levels in WT and *CmVNI2* RNAi lines. *CmUBI* was used as an internal control. Plant height (**c**) and flower diameter (**d**) of WT and *CmVNI2* RNAi lines. **e** Heatmap of the changes in differentially accumulated flavonoids (DAFs) in flowers of WT and *CmVNI2* RNAi lines. Red indicates increase, while blue indicates decrease. **f**–**i** Accumulation of flavonols, flavanones, flavone glycosides, and flavone aglycones in flowers of WT and *CmVNI2* RNAi lines. dw, dry weight. *CmVNI2* RNAi1 and *CmVNI2* RNAi2 are two independent *CmVNI2* RNAi lines. Values are means ± SDs. Standard error bars were calculated from three biological replicates, which were sampled as described previously. Asterisks indicate significant differences between WT and *CmVNI2* RNAi lines (Student’s *t*-test; *P < 0.05; **P < 0.01; ***P < 0.001)

To characterize the role of *CmVNI2* in flavonoid biosynthesis, RNA interference (RNAi) was used to knock down *CmVNI2* in wild chrysanthemum (*C. indicum*) (Fig. 2a). qRT-PCR analysis confirmed significantly lower expression levels of CmVNI2 in *CmVNI2* RNAi lines compared with wild type (WT) plants (Fig. 2b). *CmVNI2* RNAi lines exhibited significantly reduced plant height relative to WT, while flower diameter showed no significant differences between WT and RNAi lines (Fig. 2c, d). To further examine the effects of *CmVNI2* RNAi on flavonoid accumulation, flavonoid-targeted metabolite profiling analysis was performed, quantifying 200 flavonoid metabolites in flowers of both WT and two *CmVNI2* RNAi lines using an UPLC-ESI–MS/MS platform (Supplementary Datasets S2–S6). Principal component analysis distinctly separated WT from *CmVNI2* RNAi lines, with the three biological replicates of each line clustering together (Fig. S3a). The analysis identified 55 and 56 differentially accumulated flavonoids (DAFs; fold change > 2, *P*-value ≤ 0.05) in comparisons between WT vs. *CmVNI2* RNAi1 and WT vs. *CmVNI2* RNAi2, respectively, including 15 upregulated and 40 downregulated flavonoids in WT vs. *CmVNI2* RNAi1, along with 16 upregulated and 40 downregulated flavonoids in WT vs. *CmVNI2* RNAi2 (Fig. S3b; Supplementary Dataset S3). DAF analysis revealed significantly decreased contents of flavonol, flavanone, and flavone glycosides in *CmVNI2* RNAi flowers (Fig. 2e–h). Conversely, the total content of flavone aglycones (apigenin, diosmetin, hispidulin, luteolin, acacetin, and scutellarein) showed significant increases in these flowers (Fig. 2i).

**Fig. 3:**
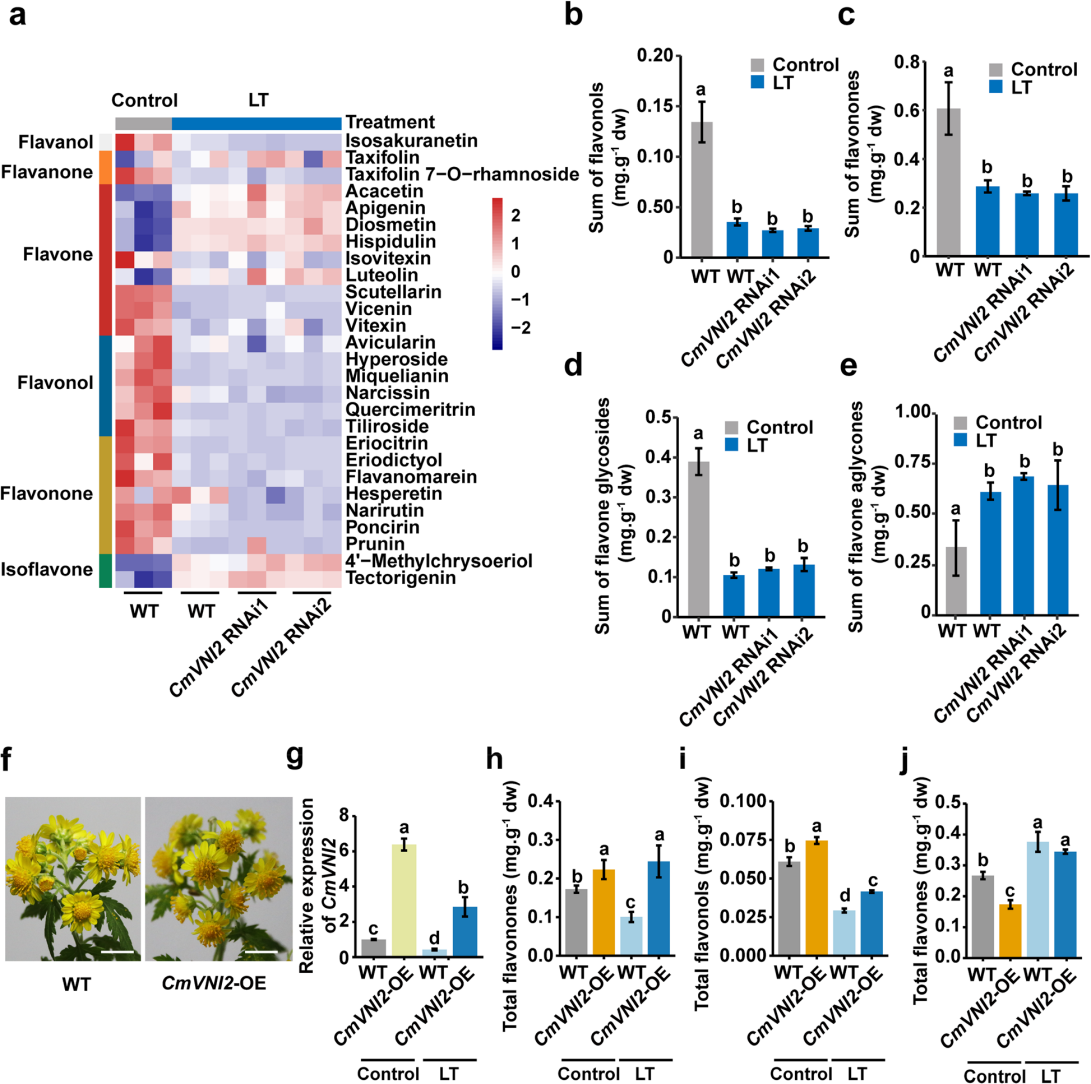
Flavonoid contents in flowers of WT and *CmVNI2* RNAi lines under normal and low temperatures. **a** Heatmap of the DAF changes in flowers of WT and *CmVNI2* RNAi lines under control and low-temperature conditions. Control, at 22 °C; LT, after 7-day low-temperature exposure at 10 °C. Red indicates increase, while blue indicates decrease. **b**–**e** Contents of flavonols, flavonones, flavone glycosides, and flavone aglycones in flowers of WT and *CmVNI2* RNAi lines under control and low-temperature conditions. dw, dry weight. **f** Morphological characteristics of WT and *CmVNI2*-OE flowers. Scale bar: 1 cm. **g** qRT-PCR analysis of *CmVNI2* transcript levels in WT and *CmVNI2*-OE flowers under control and low-temperature conditions. *CmUBI* was used as an internal control. **h**–**j** Contents of total flavonones (hesperetin and naringoside), total flavonols (quercetin and kaempferol), and total flavones (apigenin, luteolin, acacetin, diosmetin, and cynaroside) in WT and *CmVNI2*-OE flowers under control and low-temperature conditions. dw, dry weight. Data are presented as means ± SDs (*n* = 3). Significantly different values (*P* < 0.05) are indicated with lowercase letters. Values were calculated by one-way ANOVA followed by Tukey’s HSD test

To examine the impact of low temperature on CmVNI2-regulated flavonoid accumulation, we analyzed the flavonoid contents in WT and *CmVNI2* RNAi flowers under controlled growth conditions and following a 7-day low-temperature treatment (Fig. 3a; Supplementary Dataset S7). Flavonol and flavanone levels, which were reduced in *CmVNI2* RNAi lines, showed significant reduction in WT plants after 7-day low-temperature treatment compared with WT under controlled growth conditions (Fig. 3b, c). Furthermore, the content of flavone glycosylated derivatives significantly decreased in flowers of both WT and *CmVNI2* RNAi lines following low-temperature treatment (Fig. 3d). In contrast, the total content of flavone aglycones increased significantly in both *CmVNI2* RNAi lines and WT plants after low-temperature exposure relative to WT under controlled growth conditions (Fig. 3e).

To validate the function of CmVNI2 in regulating flavonoid metabolite redistribution under low-temperature conditions, we overexpressed (OE) *CmVNI2* in *C. indicum* flowers. The flower phenotype remained unchanged between WT and *CmVNI2*-OE flowers (Fig. 3f). qRT-PCR analysis revealed significantly elevated expression levels of *CmVNI2* in *CmVNI2*-OE flowers compared with WT under both controlled growth conditions and low-temperature exposure (Fig. 3g). Under controlled growth conditions and after 7-day low-temperature treatment, the contents of total flavonones (hesperetin and naringoside) and total flavonols (quercetin and kaempferol) increased significantly in *CmVNI2*-OE flowers compared with WT (Fig. 3h, i; Supplementary Dataset S8). However, total flavones (apigenin, luteolin, acacetin, diosmetin, and cynaroside) significantly decreased in *CmVNI2*-OE flowers under controlled growth conditions, while showing no significant changes in *CmVNI2*-OE flowers under low-temperature treatment compared with WT (Fig. 3j; Supplementary Dataset S8). Thus, *CmVNI2* functions as a regulator of flavonoid metabolic flux in response to low temperatures.

### *CmVNI2* knockdown affects the expression of genes related to flavonol biosynthesis

**Fig. 4:**
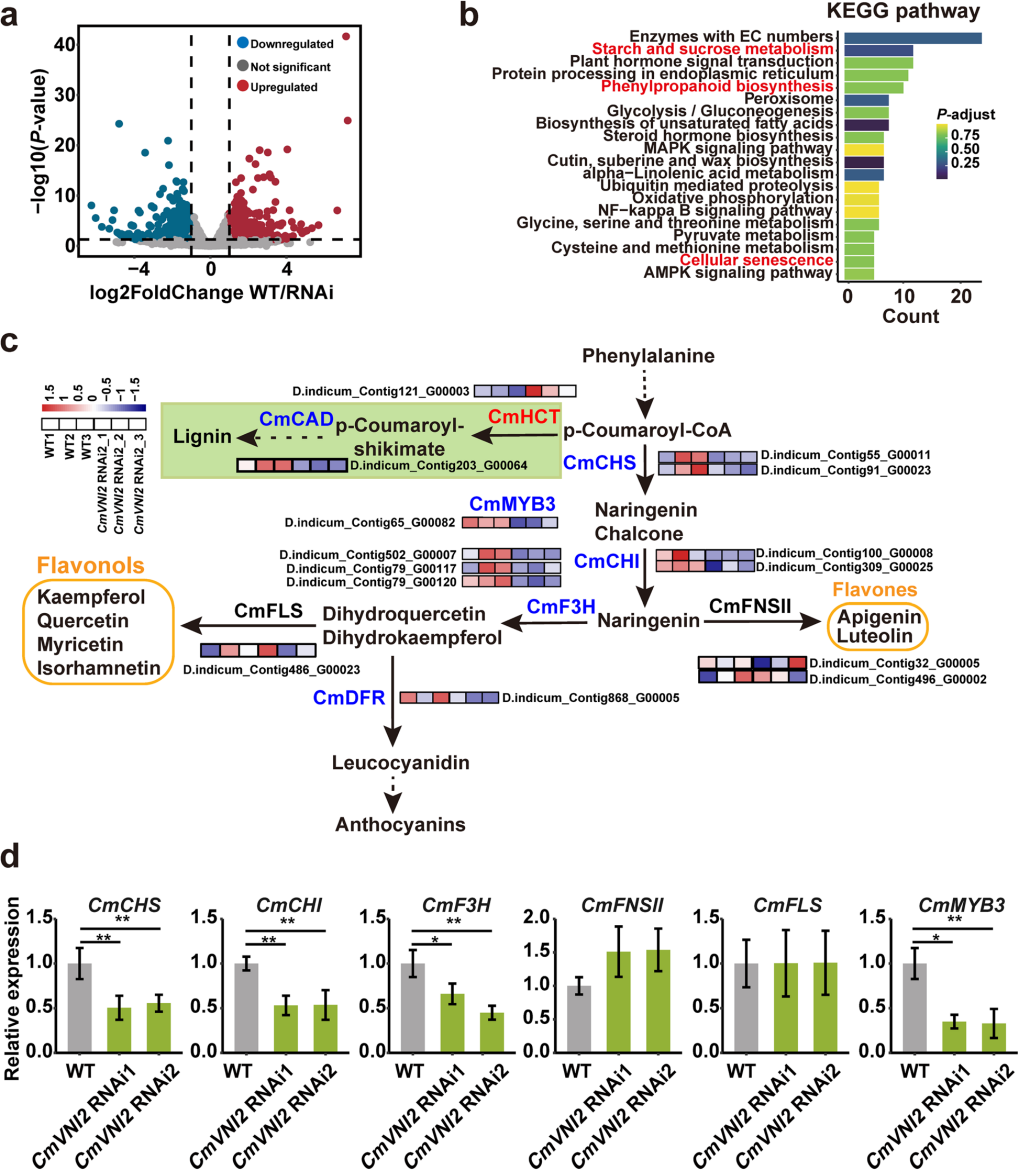
CmVNI2 influences the expression of genes involved in flavonol biosynthesis. **a** Differential expression analysis of genes between WT and *CmVNI2* RNAi lines. **b** KEGG pathways significantly enriched in *CmVNI2* RNAi lines. **c** Flavonoid biosynthesis pathway in chrysanthemum. Red indicates upregulated DEGs, while blue indicates downregulated DEGs. **d** Expression validation by qRT-PCR. *CmUBI* served as an internal control. Data are presented as means ± SDs (*n* = 3). Asterisks indicate statistically significant differences (Student’s *t*-test; *P < 0.05; **P < 0.01; ***P < 0.001)

To investigate the regulatory network associated with CmVNI2, we performed RNA-seq experiments to analyze the transcriptomes of flowers from WT and *CmVNI2* RNAi lines. The analysis identified 1,606 differentially expressed genes (DEGs) using a threshold of |log_2_ fold change|≥ 1 and *P*-value ≤ 0.05. Of these DEGs, 782 were upregulated and 824 were downregulated in *CmVNI2* RNAi transcriptomes compared with WT (Fig. 4a; Supplementary Dataset S9). Kyoto Encyclopedia of Genes and Genomes (KEGG) pathway analysis revealed that the DEGs were primarily enriched in “starch and sucrose metabolism”, “phenylpropanoid biosynthesis”, and “cellular senescence” (Fig. 4b). Gene Ontology (GO) enrichment analysis showed that the downregulated DEGs were significantly associated with the terms “cell wall”, “pollen development”, and various metabolic processes, such as “flavonoid biosynthetic process” and “acyl-CoA metabolic process” (Fig. S4). Functional annotation identified nine downregulated DEGs related to flavonoid biosynthesis, comprising two *CmCHS*, two *CmCHI*, three *CmF3H*, one *CmDFR*, and one flavonol-specific MYB TF, *CmMYB3*, along with two DEGs involved in lignin biosynthesis: upregulated *CmHCT* and downregulated *cinnamyl alcohol dehydrogenase* (*CmCAD*) (Fig. 4c). Notably, *CmCHS, CmCHI*, and *CmF3H* are structural genes encoding enzymes essential for flavonol biosynthesis. Furthermore, *CmMYB3* (*C. indicum*_Contig65_ G00082) exhibited significant downregulation in the *CmVNI2* RNAi line compared with WT, suggesting its role as a transcriptional target of *CmVNI2* (Fig. 4c, d). No significant changes were observed in the transcription levels of *CmFLS* and *CmFNSII* between WT and *CmVNI2* RNAi lines (Fig. 4c). qRT-PCR analysis confirmed the RNA-seq findings by assessing the abundance of flavonol biosynthesis gene transcripts in flowers from both WT and *CmVNI2* RNAi lines (Fig. 4d). The expression patterns obtained from both methods were consistent.

### Genome-wide identification of CmVNI2 binding sites

**Fig. 5:**
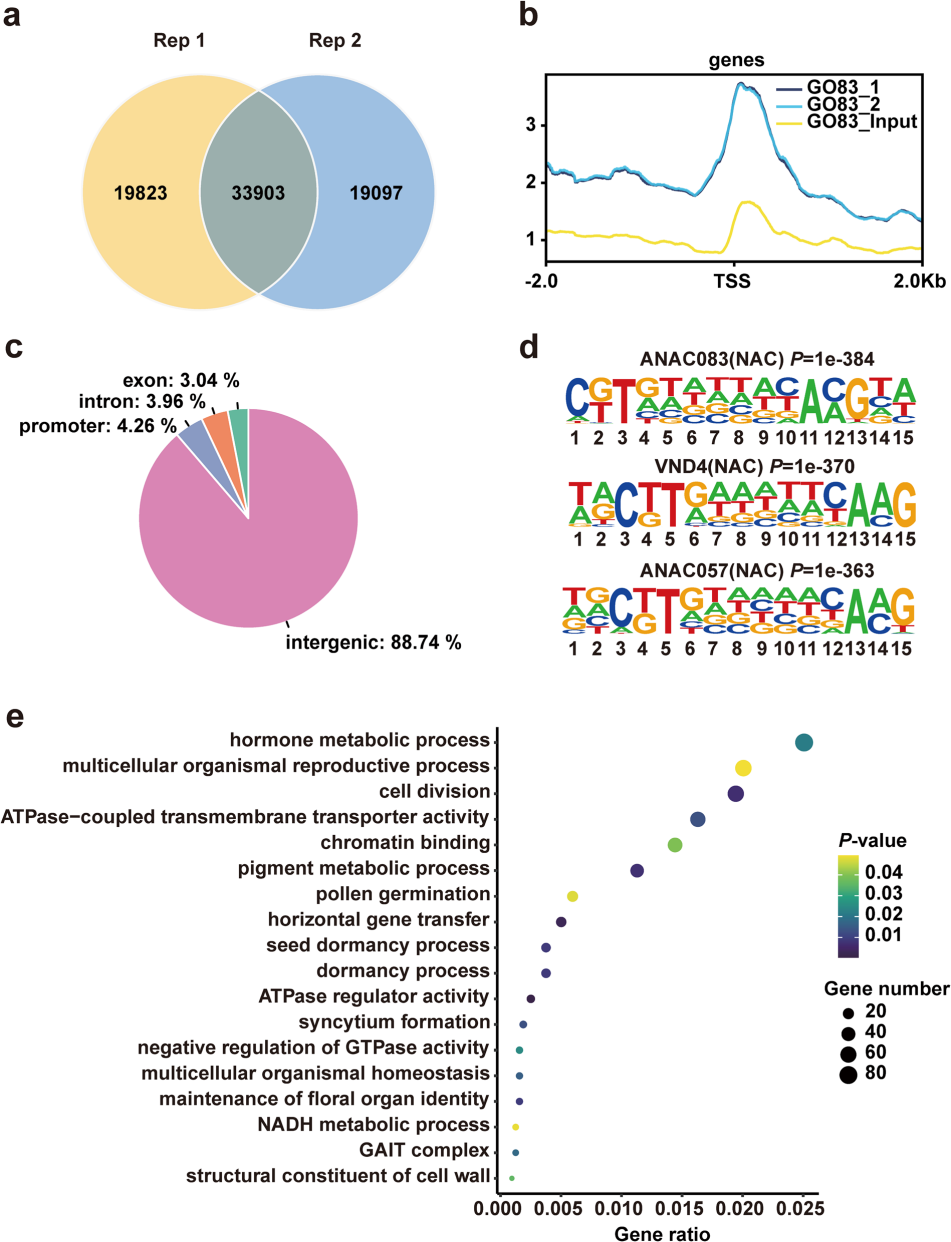
Genome-wide identification of CmVNI2 binding sites by DAP-seq. **a** Identification of high-confidence CmVNI2 binding peaks from two DAP-seq replicates. **b** Read distribution within the 2-kb sequence flanking the TSS. **c** Distribution of CmVNI2 binding peaks across genomic features. **d** DNA motifs enriched among CmVNI2 DNA-binding sites, determined by HOMER. *P*-values are indicated. **e** Top enriched GO terms of CmVNI2-bound genes identified by DAP-seq. Larger dots indicate higher gene counts

To elucidate the transcriptional regulation of flavonoid biosynthesis by CmVNI2, DNA affinity purification sequencing (DAP-seq) was conducted to identify CmVNI2 binding sites across the genome. Details of the total number of clean sequencing reads and mapping rates for each replicate are provided in Supplementary Table S1. The analysis identified 33,903 enriched peaks, corresponding to 3,709 genes in two biological replicates, establishing these as high-confidence binding regions for CmVNI2 (Fig. 5a; Supplementary Datasets S10–S12). These binding sites, ranging from 239 to 3,152 bp in length, were predominantly located near transcriptional start sites (TSSs) (Fig. 5b; Fig. S5). Analysis of the binding profiles revealed CmVNI2 binding distribution across exons (3.04%), introns (3.96%), intergenic regions (88.74%), and promoter regions (4.26%) within 2 kb upstream from a TSS (Fig. 5c). Investigation of CmVNI2 DNA-binding properties by de novo motif prediction using HOMER revealed significantly enriched known motifs. NAC motifs, including ANAC083, VND4, and ANAC057, were prevalent, accounting for 4,416, 3,772, and 3,533 CmVNI2 binding peaks, respectively (Fig. 5d). GO enrichment analysis of genes containing CmVNI2-binding sites showed significant overrepresentation in categories including “hormone metabolic process”, “cell division”, “pigment metabolic process”, “pollen germination”, and “structural constituent of cell wall” (Fig. 5e). These findings suggest CmVNI2’s direct regulatory role in various biological pathways associated with cell wall structure and metabolic biosynthesis, indicating its involvement in multiple cellular processes.

### Identification of direct target genes of CmVNI2

**Fig. 6:**
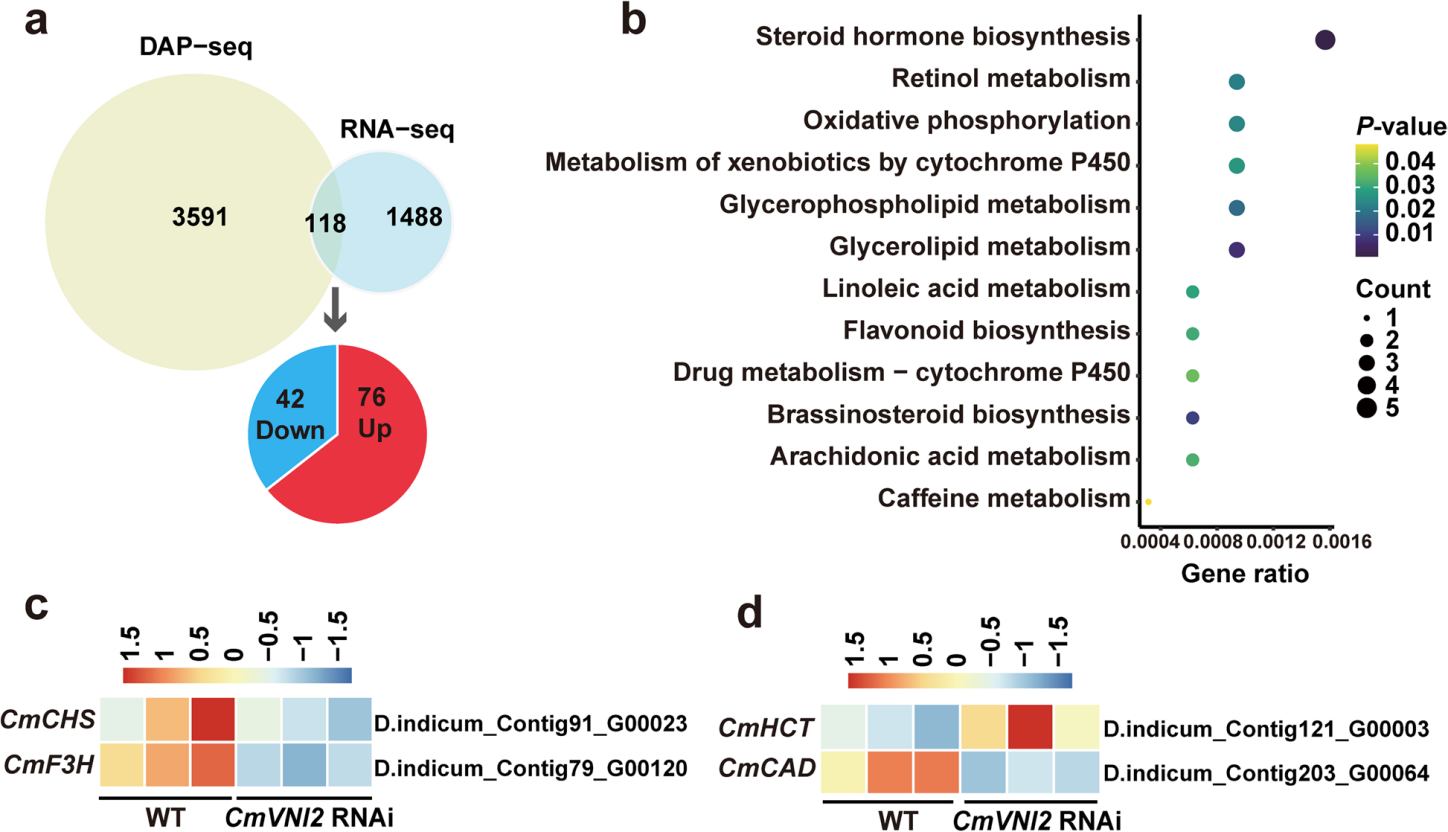
Identification of target genes of CmVNI2. **a** Venn diagram illustrating the overlap between CmVNI2-bound genes identified by DAP-seq and CmVNI2-regulated genes identified by RNA-seq. The number of genes directly downregulated or upregulated by CmVNI2 is shown. **b** Top enriched KEGG terms for the direct CmVNI2-regulated target genes. **c**–**d** Heatmap of the expression of direct CmVNI2-regulated target genes related to flavonoid and lignin biosynthesis

To identify the direct target genes of CmVNI2, we performed a comparative analysis between the 3,709 binding genes identified by DAP-seq and the 1,606 DEGs obtained from RNA-seq analysis of *CmVNI2* RNAi lines. The analysis identified 118 common genes across both datasets, indicating their status as direct targets of CmVNI2 (Supplementary Dataset S13). Of these genes, 42 (35.59%) demonstrated positive regulation by CmVNI2, showing downregulation in *CmVNI2* RNAi lines. Conversely, 76 genes (64.41%) exhibited negative regulation by CmVNI2, displaying upregulation in *CmVNI2* RNAi lines (Fig. 6a). Subsequent KEGG pathway enrichment analysis revealed that these direct target genes participate in various metabolic pathways, including “steroid hormone biosynthesis”, “retinol metabolism”, “oxidative phosphorylation”, and “flavonoid biosynthesis” (Fig. 6b). Consistent with the established function of CmVNI2 in regulating flavonol biosynthesis, *CmCHS* and *CmF3H* were identified as direct targets of CmVNI2 (Fig. 6c), suggesting that CmVNI2 regulates flavonol biosynthesis by structural genes. However, *CmMYB3* was not present in this comparative analysis. Furthermore, the analysis identified two genes involved in lignin biosynthesis: *CmHCT* and *CmCAD*, direct targets of CmVNI2 (Fig. 6d). This finding suggests that CmVNI2 influences lignin biosynthesis, thereby contributing to SCW thickening and enhancing plant acclimation to cold environments (Dong and Lin 2021).

### CmVNI2 directly regulates key genes in the flavonol biosynthetic pathway

**Fig. 7:**
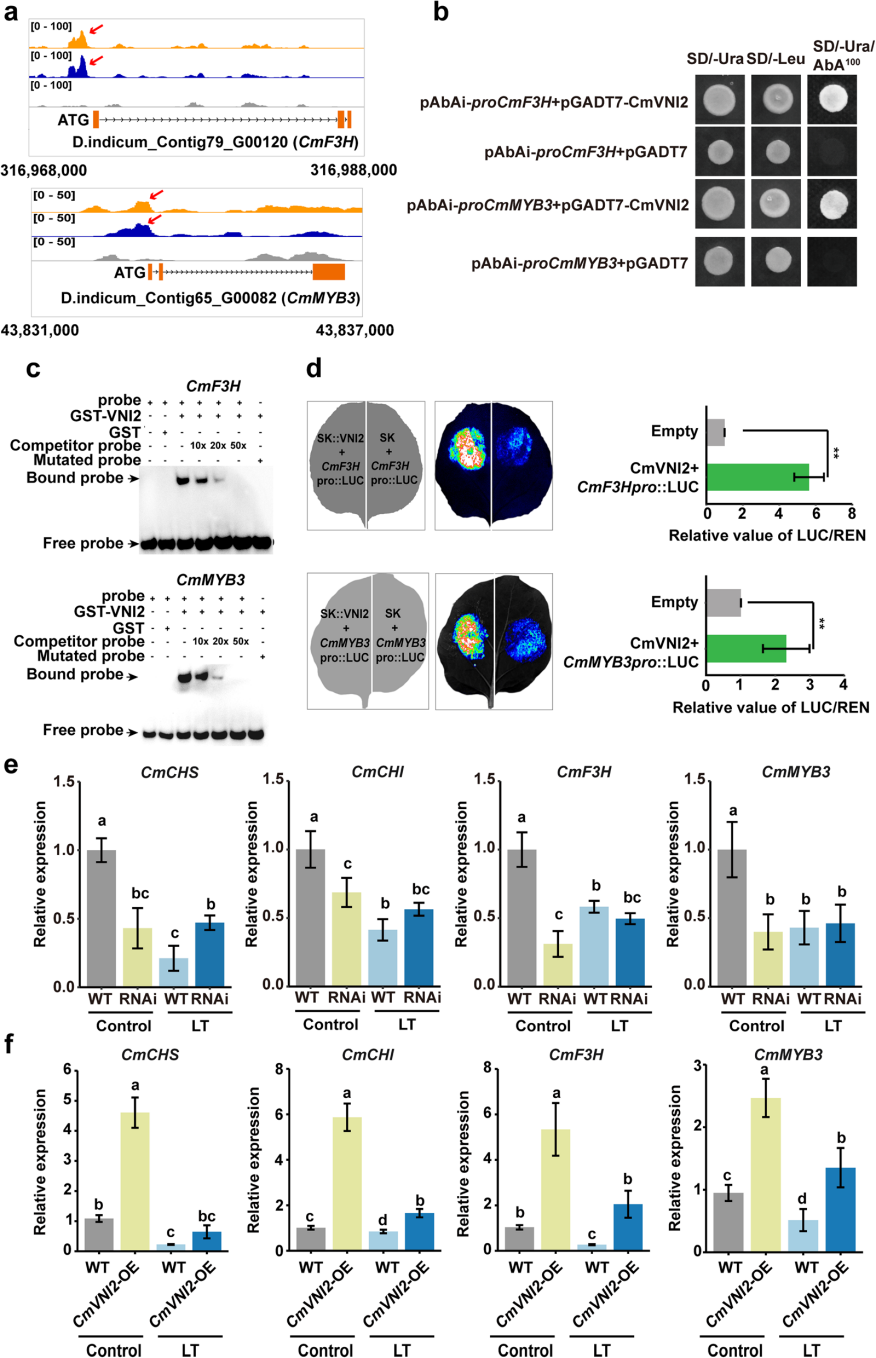
Interaction between CmVNI2 and target gene promoters. **a** CmVNI2 binding peaks (Repeats 1 and 2) and negative control (mock) over the *CmF3H* and *CmMYB3* loci as determined by DAP-seq. [0 to 100] and [0 to 50] represent the scale of the binding intensity as reflected by the height of the peak. **b** Yeast one-hybrid analysis of CmVNI2 binding to the *CmF3H* and *CmMYB3* promoters. **c** Electrophoretic mobility shift assay showing that CmVNI2 binds directly to the *CmF3H* and *CmMYB3* promoters. Purified recombinant GST-CmVNI2 (1 µg) was incubated with 2 nM biotin-labeled *CmF3H* and the *CmMYB3* promoter probe. As indicated, CmVNI2-dependent mobility shifts were detected, and competition with an unlabeled cold probe inhibited the shifts in a dose-dependent manner. **d** Bright field image and LUC activity indicating activation of the transactivation of the *CmF3H* and *CmMYB3* promoters by CmVNI2. Quantitation is shown on the right. **e** Relative expression levels of *CmCHI*, *CmCHS*, *CmF3H*, and *CmMYB3* in flowers from WT and *CmVNI2* RNAi lines under control and low-temperature conditions by qRT-PCR. Control, at 22 °C; LT, after 7-day low-temperature exposure at 10 °C. *CmUBI* was used as an internal control. **f** Relative expression levels of *CmCHI*, *CmCHS*, *CmF3H*, and *CmMYB3* in WT and *CmVNI2*-OE flowers under control and low-temperature conditions by qRT-PCR. *CmUBI* was used as an internal control. Data are presented as means ± SDs (n = 5). Asterisks indicate statistically significant differences (Student’s *t*-test; *P < 0.05; **P < 0.01; ***P < 0.001). Significantly different values (*P* < 0.05) are indicated with lowercase letters. Values were calculated by one-way ANOVA followed by Tukey’s HSD test

CmMYB3 is a crucial regulator of flavonol biosynthesis in chrysanthemum (Yang et al. 2023). RNA-seq analyses revealed significantly reduced expression of *CmMYB3* in *CmVNI2* RNAi lines compared with WT, but the *CmMYB3* locus was not present in CmVNI2 DAP-seq peaks. To investigate CmVNI2’s regulation of *CmMYB3*, the promoter sequence of *CmMYB3* was cloned and potential NAC-binding *cis*-elements were predicted using the JASPAR database (https://jaspar.genereg.net/collection/core) (Supplementary File S1). Analysis of CPM (counts per million reads) for alignments to the *CmMYB3* promoter region by DAP-seq revealed read fold enrichments of 1.974 and 2.379 in two biological replicates when compared with input control (Fig. 7a; Supplementary Table S2). These data indicate direct regulation of *CmMYB3* expression by CmVNI2. Based on the established role of SG19 MYB proteins in flavonol biosynthesis, two SG19 MYB proteins were identified in *C. indicum*: *CmMYB24_1* and *CmMYB24_2* (Fig. S6a). qRT-PCR analysis demonstrated downregulation of both *CmMYB24_1* and *CmMYB24_2* in *CmVNI2* RNAi flowers and upregulation in *CmVNI2*-OE flowers (Fig. S6b, c). Under low-temperature conditions, *CmMYB24_1* expression decreased while *CmMYB24_2* expression increased (Fig. S6b, c), suggesting that *CmMYB24_1* is a CmVNI2 target. In addition, DAP-seq and RNA-seq analyses identified two structural genes involved in flavonol biosynthesis, *CmCHS* and *CmF3H* (Fig. 7a), with CmVNI2 binding to *CHS* within its coding sequence. Yeast one-hybrid (Y1H) assays examining interactions between CmVNI2 and the promoters of *CmCHS*, *CmCHI*, *CmF3H*, *CmMYB24_1*, and *CmMYB3 *in vitro showed CmVNI2 binding to *CmF3H* and *CmMYB3* promoters but not *CmCHS*, *CmCHI*, *and CmMYB24_1* promoters (Fig. 7b; Fig. S7). Electrophoretic mobility shift assays (EMSAs) and dual-luciferase (dual-LUC) activity assays confirmed CmVNI2 binding to *CmF3H* and *CmMYB3* promoters (Fig. 7c, d). Consistent with CmVNI2’s role in regulating flavonol biosynthesis under low-temperature conditions, the expression levels of *CmCHS*, *CmCHI*, *CmF3H*, and *CmMYB3* decreased after low-temperature treatment compared with controlled growth conditions (Fig. 7e, f). These findings demonstrate that CmVNI2 directly regulates both *CmF3H* and *CmMYB3* expression in response to low temperature.

### *CmMYB3 *overexpression restores reduced flavonol accumulation caused by *CmVNI2* silencing

**Fig. 8:**
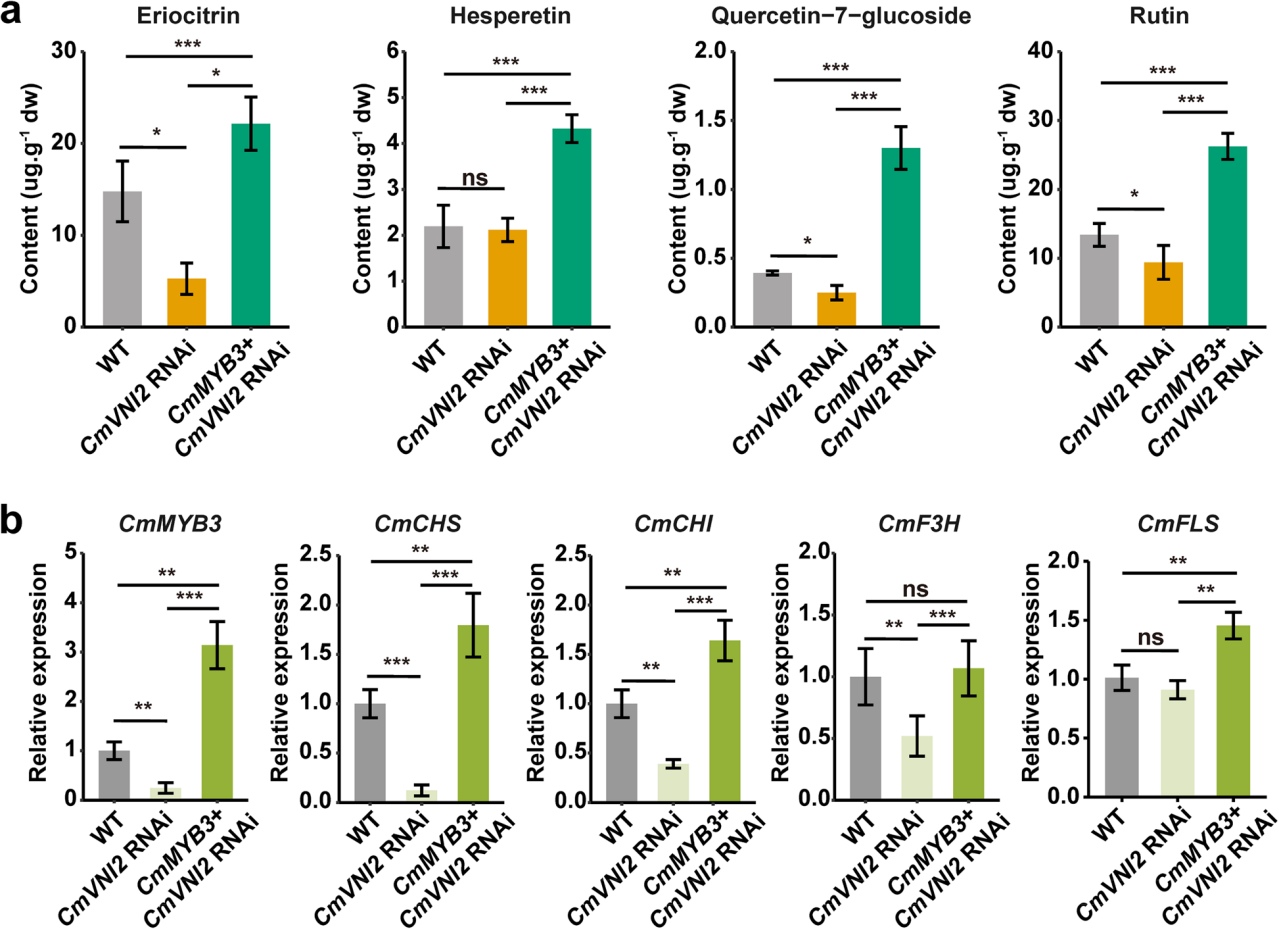
*CmMYB3* overexpression restores flavonoid biosynthesis in chrysanthemum. **a** Flavanone and flavonol contents in WT, *CmVNI2* RNAi, and *CmMYB3* + *CmVNI2* RNAi plants. dw, dry weight. **b** Relative expression levels of *CmMYB3*, *CmCHS*, *CmCHI*, *CmF3H*, and *CmFLS* in leaves from WT, *CmVNI2* RNAi, and *CmMYB3* + *CmVNI2* RNAi plants. Empty vector (GFP)-transformed leaves were used as controls. Data are presented as means ± SDs (*n* = 3). Asterisks indicate statistically significant differences (Student’s *t*-test; *P < 0.05; **P < 0.01; ***P < 0.001)

To investigate whether CmVNI2 affects flavonol accumulation by regulation of *CmMYB3*, the *CmMYB3* gene was transiently overexpressed in *CmVNI2* RNAi plants. *Agrobacterium* infiltration with *CmMYB3*-PBI121 was conducted in *CmVNI2* RNAi plants, with empty vector serving as a control in both WT and *CmVNI2* RNAi plants. To examine flavonoid content changes in *CmMYB3* + *CmVNI2* RNAi plants, leaves were collected from WT, *CmVNI2* RNAi, and *CmMYB3* + *CmVNI2* RNAi plants for quantification of marker flavonoids by UPLC-ESI–MS/MS analysis (Fig. S8). The accumulation of flavonols, including rutin and quercetin-7-glucoside, as well as flavanones such as eriocitrin and hesperetin, exhibited significant increases in *CmMYB3* + *CmVNI2* RNAi plants compared with both WT and *CmVNI2* RNAi plants. These results demonstrate that *CmMYB3* overexpression restored the reduced levels of flavonols and flavanones in *CmVNI2* RNAi plants (Fig. 8a). However, the contents of flavones such as diosmin and apigenin 7-O-glucoside; flavanones including naringenin, naringin, and prunin; and flavonols such as isorhamnetin, kaempferol, and their derivatives showed no significant differences (Fig. S9), indicating that *CmMYB3* expression predominantly influences the biosynthesis pathway of eriocitrin, hesperetin, and quercetin. qRT-PCR analysis demonstrated significantly elevated expression levels of *CmMYB3* and structural genes including *CmCHS*, *CmCHI*, *CmF3H*, and *CmFLS* in leaves from *CmMYB3* + *CmVNI2* RNAi plants (Fig. 8b). These results confirm *CmMYB3*′s involvement in the transcriptional regulation of flavonol biosynthesis mediated by CmVNI2. Together, these findings indicate that CmVNI2 regulates flavonol biosynthesis by modulating *CmMYB3* expression.

## Discussion

Flavonols significantly contribute to plant growth, development, and potentially pollen germination (Cao et al. 2024). The present study identified elevated flavonol levels in chrysanthemum disc florets containing pollen-producing stamens, suggesting flavonols’ essential role in pollen generation. F3H and FLS catalyze flavonol biosynthesis, while FNSII mediates flavone biosynthesis. Both F3H and FNSII utilize naringenin as substrate and exhibit narrow substrate specificity (Martens et al. 2003). Flavonol production requires F3H to compete with FNSII for naringenin, redirecting metabolic flux from flavone to flavonol biosynthesis. The high expression of *CmF3H* in chrysanthemum disc florets corresponds to flavonol accumulation patterns, while *CmFNSII* shows predominant expression in ray florets, correlating with flavone accumulation (Luo et al. 2022). Despite downregulation of *CHS* and *CHI*, *CmF3H* suppression prevented metabolic flux redirection toward flavonol biosynthesis, resulting in enhanced naringenin conversion to flavones (Figs. 2i and 4d). The specific expression of *CmVNI2* in disc florets emphasizes its crucial function in redirecting flavonol metabolic flux.

While limited research has addressed the role of NAC in regulating flavonoid biosynthesis, most investigations have focused on the MBW complex comprising R2R3-MYB TFs, basic helix-loop-helix TFs, and WD40 proteins (Xu et al. 2015). The Arabidopsis SG7 group of MYB regulators, including MYB11, MYB12, and MYB111, regulate flavonol accumulation by specifically activating the expression of *CHS*, *CHI*, *F3H*, and *FLS1* (Mehrtens et al. 2005). Ectopic expression of chrysanthemum SG7 R2R3 MYB regulator *CmMYB3* in tobacco and *Arabidopsis* enhances total flavonoid, quercetin, and kaempferol levels by upregulation of *AtCHS*, *AtCHI*, *AtF3H*, and *AtFLS* (Yang et al. 2023). In this study, knockdown of *CmVNI2* expression in chrysanthemum resulted in a substantial reduction in both flavanone and flavonol contents. Furthermore, the expression levels of flavonol biosynthesis-associated genes, including *CmCHS*, *CmCHI*, *CmF3H*, and *CmMYB3*, decreased in *CmVNI2* RNAi lines compared with WT. DAP-seq results revealed that CmVNI2 binds specifically to the promoters of *CmF3H* and *CmMYB3*. CmMYB3 directly binds to the promoters of both *CmCHI* and *CmFLS* genes in *C. morifolium* (Yang et al. 2023). However, *CmFLS* transcript levels showed no significant difference between *CmVNI2* RNAi lines and WT plants. Notably, *CmVNI2* overexpression in *C. indicum* flowers or *CmMYB3* within *CmVNI2* RNAi lines increased expression levels of *CmCHS*, *CmCHI*, *CmF3H*, and *CmFLS*, while reversing the reductions in flavanone and quercetin contents (Fig. 8b; Fig. S6 d). Unlike *Arabidopsis* species, no significant differences in kaempferol or isorhamnetin content were observed between plants expressing *CmVNI2* RNAi or co-expressing *CmMYB3* + *CmVNI2* RNAi, indicating distinct regulatory mechanisms across species. Recent studies have identified the SG19 group of MYB regulators as positive regulators in flavonol biosynthesis (Battat et al. 2019; Shan et al. 2020; Zhang et al. 2023). In addition, SG19 MYB proteins participate in other metabolic pathways, such as terpene biosynthesis. While SG7 MYBs directly activate *CHS*, *CHI*, *F3H*, and *FLS* expression to specifically regulate flavonol biosynthesis, SG19 MYBs primarily activate FLS gene transcription. This study revealed significant expression changes of the *SG19 R2R3-MYB CmMYB24_1* in both *CmVNI2* RNAi lines and *CmVNI2*-OE flowers, suggesting that *CmMYB24_1* is a downstream target of CmVNI2 in chrysanthemum. Further research is necessary to elucidate the interaction between CmVNI2 and *CmMYB24_1*. The presented evidence indicates that CmVNI2 exercises both direct and indirect control over flavonoid biosynthesis in chrysanthemum flowers. Thus, the regulatory role of CmVNI2 in flavonol biosynthesis primarily involves direct activation of *CmF3H* and *CmMYB3*, two essential genes that influence flavonol biosynthesis by redirecting metabolic flux and modulating *CmFLS* expression.

Lignin has been extensively documented as a contributor to SCW thickening and plant responses to various abiotic stresses (Cesarino 2019). The NAC–MYB-based gene regulatory network has been established to regulate SCW formation, including lignin biosynthesis (Nakano et al. 2015). In *Arabidopsis*, AtVNI2 provides a molecular link between plant response and environmental stress, influencing leaf longevity and exerting a negative regulatory effect on xylem vessel formation (Yamaguchi et al. 2010). In the present study, the expression of lignin biosynthesis genes, such as *HCT* and *CAD*, was directly regulated by CmVNI2, demonstrating its role in lignin biosynthesis. HCT and CHS are two key enzymes that direct metabolic flux toward either the lignin or flavonoid pathway. Our findings demonstrate that the regulation of these two genes by CmVNI2 is opposite, indicating that CmVNI2 may mediate a trade-off between lignin and flavonoid biosynthesis. Collectively, these results reveal a regulatory network governing both lignin and flavonoid pathways, which constitute two important branches of phenylpropanoid metabolism.

Various environmental factors, including both biotic and abiotic stresses, can influence flavonoid accumulation by regulating the expression of structural or regulatory genes. The resulting accumulation of flavonoids serves to protect plants against these stresses. In experiments with *Arabidopsis*, significant amounts of the flavonol quercetin-3Rha-7Rha were detected in all samples after 14 days of cold acclimation at 4 °C; however, it was undetectable in some WT *Arabidopsis* plants maintained at room temperature (Schulz et al. 2015). In contrast to these findings, this study revealed that the contents of flavonols, flavanones, and flavone glycosides in chrysanthemum flowers decreased after 7-day low-temperature exposure at 10 °C. The differing patterns of flavonoid accumulation between *Arabidopsis* and chrysanthemum in response to low temperatures may be attributed to two primary factors. First, flavones are absent in *Arabidopsis* flowers but are abundant in chrysanthemum flowers (Martens and Mithöfer 2005; Hao et al. 2022). Second, the plant materials used in this study were reproductive organs (flowers), whereas vegetative organs (leaves) were examined in *Arabidopsis*. Vegetative and reproductive organs exhibit contrasting patterns of flavonoid accumulation when exposed to low temperatures (Mao et al. 2022).

In the plant kingdom, flavonoids are typically stored in vacuoles as glycosides. Glycosylation stabilizes reactive aglycones through the addition of sugar groups on carbon and hydroxyl moieties for subsequent activation and release. Compared with their glycoside forms, corresponding aglycones often exhibit greater resistance or enhanced tolerance to oxidative stress (Le Roy et al. 2016; Sudheeran et al. 2020). The research revealed that the accumulation of flavone glycosides significantly decreased in both WT and *CmVNI2* RNAi lines under low-temperature conditions. Conversely, the levels of flavone aglycones, including apigenin, diosmetin, hispidulin, luteolin, acacetin, and scutellarein, increased in both WT and *CmVNI2* RNAi lines following low-temperature treatment. Thus, flavone aglycones may function to mitigate reactive oxygen species under low-temperature stress. These findings indicate that flavone aglycones are predominant among the flavonoids in chrysanthemum flowers and provide protective benefits against low-temperature stress.

## Materials and methods

### Plant materials

The chrysanthemum cultivar (*C. morifolium* cv. Yutai) used in this study was propagated by cuttings. Shoots with at least one node were harvested from stock plants, placed in a mixture of 1:1 (v/v) peat and vermiculite, and grown in a greenhouse maintained at 23 ± 1 °C and 40% relative humidity. After two weeks, when the cuttings had taken root, they were transplanted into 10-cm-diameter pots containing soil and grown in a greenhouse.

The wild chrysanthemum (*C. indicum*) used in this study was propagated by in vitro culture. Short stems with at least one node were cultured on media comprising ½ Murashige and Skoog medium for 40 days, transplanted into 9-cm-diameter pots containing a mixture of 1:1 (v/v) peat and vermiculite, and subsequently grown in a culture room at 23 ± 1 °C, 40% relative humidity, and 100 μmol m^−2^ s^−1^ illumination with fluorescent lamps.

### Gene isolation and phylogenetic analysis

The *CmVNI2* open reading frame sequence was obtained from a chrysanthemum RNA-seq dataset, and the full-length sequence was amplified using PrimeSTAR Max DNA polymerase (TaKaRa, Tokyo, Japan) according to the manufacturer’s protocol. The deduced full-length amino acid sequence of CmVNI2 was aligned with NAC protein sequences from various species using BioEdit (http://www.mbio.ncsu.edu/BioEdit/bioedit.html) and ClustalW (http://www.ch.embnet.org/software/ClustalW.html). Phylogenetic analysis was performed using MEGA v5 and the maximum likelihood method with 1,000 bootstrap replicates.

### RNA isolation and qRT-PCR analysis

Total RNA was extracted from chrysanthemum flowers using the Plant RNA Extraction Kit (TaKaRa). First-strand cDNAs were synthesized from 1 µg of total RNA using the PrimeScript™ RT Master Mix (TaKaRa) according to the manufacturer’s protocol. qRT-PCR (20 μl containing 1 μl of cDNA) was performed using the StepOne Real-Time PCR System (Applied Biosystems, Waltham, MA, USA) with TB Green Fast qPCR Mix (TaKaRa). The chrysanthemum ubiquitin gene (GenBank accession NM_112764) served as an internal control. A minimum of three biological replicates were performed for all experiments. Primers used for qRT-PCR analysis are listed in Supplementary Table S3.

### Chrysanthemum transformation

To construct the *CmVNI2* RNAi plasmid, 200-bp sense and antisense *CmVNI2* fragments were amplified using the primers shown in Supplementary Table S1. The fragments were subsequently cloned and inserted into the pFGC1008 vector, which was then used to suppress *CmVNI2* expression. Plants were transformed using *Agrobacterium* strain EHA105-mediated infiltration of leaf discs, as previously described (Liu et al. 2023). Hygromycin-resistant primary transformants were screened by PCR using the primers listed in Supplementary Table S3 to confirm the presence of the transgene.

### Metabolite profiling and data processing

A total of 250 mg of flowers from *CmVNI2* RNAi lines (9–1 and 9–2) and WT plants (three independent plants of each line) was used. The samples were prepared and extracted for LC–MS as previously described (Luo et al. 2022). Briefly, freeze-dried samples were crushed into powder and extracted overnight at 4 °C with 70% aqueous methanol containing 0.1 mg/L lidocaine as an internal standard. After centrifugation at 10,000 × *g* for 10 min, the supernatant was filtered, and 2 μl of the filtrate was injected into the UPLC-ESI–MS/MS system. The targeted compounds were analyzed using an Acquity UPLC I-Class System equipped with a binary solvent manager, a sample manager with a flow-through needle, and an Acquity UPLC CSH C18 RP column (150 × 2.1 mm, particle size 1.7 μm) coupled to a triple quadrupole mass spectrometer (Xevo TQ-S MS), all from Waters Corp., Milford, MA, USA. Data filtering, peak detection, alignment, and calculations were performed using MassLynx (v4.1; Waters Corp.). Metabolites were identified by comparing the m/z values, retention times, and fragmentation patterns with standards and by searching internal and public databases.

### RNA-seq analysis

Total RNA was extracted from the flowers of *CmVNI2* RNAi lines (RNAi1 and RNAi2) and WT plants (three independent plants of each line) using the TaKaRa MiniBEST Plant RNA Extraction Kit. Nine RNA-seq libraries were constructed and sequenced on the Illumina sequencing platform by MetWare Biotechnology Co., Ltd., Wuhan, China. The workflow for RNA-seq data analysis followed previously described methods (Luo et al. 2022). Briefly, raw reads obtained from sequencing machines were filtered to remove reads containing adapters, unknown nucleotides (> 10%), and low-quality (Q-value of 20) bases. The high-quality clean reads were then mapped to the reference genome using Bowtie. Gene abundance was normalized to RPKM (reads per kb per million reads). Differential expression analysis between WT and RNAi lines was performed using the edgeR package (fold change ≥ 2 and false discovery rate < 0.05). GO annotation was performed with PANNZER2. The enrichment test was performed with ClusterProfiler.

### DAP-seq analysis

DAP-seq was performed following established protocols. Briefly, a *C. indicum* genomic DNA library was prepared using the NEBNextDNA Library Prep Master Mix Set Kit for Illumina (NEB, Ipswich, MA, USA). The Halo-CmVNI2 vector was generated by incorporating the full-length open reading frame of *CmVNI2* into the PFN19 K HaloTaq T7 SP6 Flexi Vector. The Halo-CmVNI2 recombinant protein was expressed following the instructions of the TNT SP6 Coupled Wheat Germ Extract System (Promega, Fitchburg, WI, USA). The recombinant proteins were attached to Halo-Tag ligand-coupled magnetic beads (Promega), purified with 50 µl of equilibration buffer, verified by Western blotting, and quantified using semiquantitative dot blot analysis. The DAP genomic DNA library was then incubated with the Halo-CmVNI2 protein at room temperature for 1 h. The DNA specifically bound to the Halo-CmVNI2 protein was eluted, recovered, and amplified by PCR. The enriched DNA underwent sequencing using an Illumina NovaSeq 6000 instrument at Biorun Biotechnology Co., Ltd., Wuhan, China.

After Illumina sequencing, the clean DAP-seq data were mapped to the *C. indicum* genome using Burrows–Wheeler aligner (v0.7.17-r1188) (Li and Durbin 2010; Deng et al. 2024). Subsequently, peak-calling analysis was performed to identify DNA fragments interacting with TFs across the genome. All reads within 2 kb upstream and downstream of the TSS and transcription end site were measured using deepTools (v3.5.12.0) (Ramírez et al. 2016). Peaks with q-values < 0.05 were identified using MACS2 (v2.2.7.1) (Zhang et al. 2008). The peak-related genes were annotated in BEDTools (v2.30.0) (Quinlan and Hall 2010). HOMER (Hypergeometric Optimization of Motif EnRichment, v4.11; http://homer.ucsd.edu/homer/motif/index.html) was used for motif detection (Heinz et al. 2010). Furthermore, functional clustering by KEGG and GO analyses was performed to examine the combined DNA features and functions. Two biological replicates were analyzed, with the obtained genomic DNA serving as the control.

### Y1H assays

Y1H assays were performed using the Matchmaker Gold Yeast One-Hybrid Library Screening System (Clontech, San Jose, CA, USA) according to the manufacturer’s protocol. The coding sequence of *CmVNI2* was amplified from *C. indicum* cDNA and inserted into the pGADT7 vector (Clontech) to create pGADT7-*CmVNI2*. Simultaneously, the promoter sequences (200 bp) of *CmF3H* and *CmMYB3* containing CmVNI2 binding sites were isolated from *C. indicum* genomic DNA and inserted into the pAbAi vector. The resulting pAbAi vector containing target gene sequences was transformed into the yeast strain Y1H Gold. Transformants were cultured on solidified dextrose (SD)/− Ura agar plates for 2–3 days at 30 °C. Positive colonies were subsequently grown on SD/− Ura agar medium containing various abscisic acid (AbA) concentrations to determine appropriate concentration and eliminate self-activation. Positive colonies were then transformed with pGADT7-*CmVNI2* and cultured on SD/− Leu media containing appropriate AbA concentration. pAbAi-*pro53* and empty pGADT7 served as positive and negative controls, respectively. Vector construction primers are listed in Supplementary Table S3.

### EMSA

The complete CmVNI2 coding sequence was cloned and inserted into the pGEX-4 T-2 vector to generate pGEX-4 T-CmVNI2. This vector was subsequently transformed into the *Escherichia coli* strain BL21(DE3). To obtain soluble GST-CmVNI2 proteins, *E. coli* containing pGEX-4 T-CmVNI2 was cultured at 28 °C for 16 h with 0.2 mM isopropyl-β-D-thiogalactopyranoside. The recombinant GST-CmVNI2 fusion protein was isolated from *E. coli* and purified using Glutathione Sepharose 4B (GE Healthcare, Pittsburgh, PA, USA) according to the manufacturer’s protocol. The *CmMYB3* promoter probe was synthesized and labeled with biotin. Unlabeled and mutated biotin probes for the *CmMYB3* promoter served as competitors. EMSAs were performed using the Chemiluminescent Nucleic Acid Detection Module Kit (Thermo Fisher Scientific, Waltham, MA, USA). Briefly, the probes were incubated with nuclear extract at room temperature for 30 min. The reaction mixture was separated on a nondenaturing 0.5 × TBE 6% polyacrylamide gel for 1 h at 60 V at 4 °C and transferred onto Biodyne® B nylon membranes (Pall Corp., Washington, NY, USA). The signals were detected using kit reagents and ChemiDoc XRS (Bio-Rad Laboratories, Hercules, CA, USA). The oligonucleotide probes utilized are listed in Supplementary Table S3.

### Dual-LUC reporter assay

The complete coding sequences of *CmVNI2* were cloned and inserted into the pGreen II 62-SK vector following the manufacturer’s protocol. The *CmMYB3* promoter fragment was inserted upstream of *firefly luciferase* (*LUC*) in the pGreen II 0800-LUC vector, which constitutively expresses *Renilla luciferase* (*REN*) under the cauliflower mosaic virus 35S promoter. Both plasmids were transfected into *Nicotiana benthamiana* leaves using *Agrobacterium tumefaciens*-mediated infiltration (strain GV3101). After three days, the tobacco leaves injected with *A. tumefaciens* were treated with a reaction mixture containing 50 mg/L luminal substrate D-luciferin on the abaxial surface, followed by a 5-min reaction in darkness. Luciferase activity images were captured using a CCD camera (CHEMIPROHT 1300B/LND, 16 bits; Roper Scientific, Sarasota, FL, USA). The ratios of firefly and *Renilla* luciferase activities were measured using dual-LUC assay reagents (Promega, Madison, WI, USA). The primers used for the LUC assay are listed in Supplementary Table S3.

### Transient overexpression in chrysanthemum seedlings

The full-length coding sequence of *CmVNI2* or *CmMYB3* was inserted into the vector and transformed into *A. tumefaciens* cells (strain GV3101). The chrysanthemum florets or seedlings were immersed in *A. tumefaciens* suspension for transient overexpression of *CmVNI2* or *CmMYB3*, followed by infiltration under vacuum at 1.0 MPa. The seedlings were first grown in dark for 12 h after infiltration and then transferred to light. After 3 days, the samples were immediately frozen in liquid nitrogen at − 80 °C until use.

## Supplementary Information

Supplementary Material 1: Supplementary Figure S1. Sequence alignment of NAC proteins. Supplementary Figure S2. Phylogenetic relationships of CmVNI2 and NACs with known function. Supplementary Figure S3. Flavonoid-targeted metabolite profiling analysis in flowers of WT and CmVNI2 RNAi lines. Supplementary Figure S4. Results of Gene Ontology biological process term enrichment of downregulated DEGs. Supplementary Figure S5. Analysis of the CmVNI2 binding site length. Supplementary Figure S6. CmVNI2 influences the expression of SG19 MYBs and CmFLS. Supplementary Figure S7. Yeast one-hybrid analysis of CmVNI2 binding to the promoters of flavonol biosynthesis-related genes. Supplementary Figure S8. Flavonoid analysis of CmMYB3 overexpression in chrysanthemum using an UPLC-ESI–MS/MS system. Supplementary Figure S9. Flavonoid contents in CmMYB3 overexpression plants. Supplementary File S1. CmMYB3 promoter sequences. Supplementary Table S1. Number of total clean sequencing reads and mapping rate for each replicate of DAP-seq. Supplementary Table S2. Read count and fold enrichment in the fragment of the CmMYB3 promoter for each replicate of DAP-seq. Supplementary Table S3. List of primers used in this study.

Supplementary Material 2: Supplementary Dataset S1. Co-expression analysis between transcriptional factors and flavonoid biosynthesis genes. Supplementary Dataset S2. Flavonoid-targeted metabolic analysis in flowers from WT and CmVNI2 RNAi lines. Supplementary Dataset S3. Statistics of differentially accumulated flavonoids in flowers between WT vs CmVNI2 RNAi lines. Supplementary Dataset S4. Information of targeted flavonoid compounds. Supplementary Dataset S5. Calibration method for quantifying flavonoid content. Supplementary Dataset S6. Recovery and relative standard deviation of flavonoid quantification. Supplementary Dataset S7. Flavonoid-targeted metabolic analysis in flowers from WT and CmVNI2 RNAi lines treated with low temperature. Supplementary Dataset S8. Flavonoid-targeted metabolic analysis in WT and CmVNI2-OE flowers.

Supplementary Material 3: Supplementary Dataset S9. List of differentially expressed genes from CmVNI2 RNAi lines by RNA-seq. Supplementary Dataset S10. List of DAP-seq enriched peaks in biological replicate 1. Supplementary Dataset S11. List of DAP-seq enriched peaks in biological replicate 2. Supplementary Dataset S12. List of DAP-seq enriched peaks in both biological replicates. Supplementary Dataset S13. List of CmVNI2-regulated direct target genes.

## Data Availability

Data will be available from the corresponding author upon reasonable request.

## Abbreviations

*CAD*: Cinnamyl alcohol dehydrogenase
*CHS*: Chalcone synthase
*CHI*: Chalcone-flavanone isomerase
*CPM*: Counts per million reads
*DAFs*: Differentially accumulated flavonoids
*DAP-seq*: DNA Affinity Purification Sequencing
*DEGs*: Differentially expressed genes
*DFR*: Dihydroflavonol 4-reductase
*EMSA*: Electrophoretic mobility shift assays
*FLS*: Flavonol synthase
*GO*: Gene Ontology
*HCT*: Hydroxycinnamoyl-CoA shikimate/Quinate hydroxycinnamoyl transferase
*KEGG*: Kyoto Encyclopedia of Genes and Genomes
*LUC*: Luciferase
*NAC*: No apical meristem–Arabidopsis transcription activation factor–cup-shaped cotyledon
*OE*: Overexpressed
*RNAi*: RNA interference
*RNA-seq*: RNA sequencing
*qRT-PCR*: Quantitative real-time PCR
*SG7 MYB*: R2R3-MYB subgroup seven transcription factors
*SCW*: Secondary cell wall
*TSSs*: Transcriptional start sites
*VNI2*: VND-INTERACTING2
*WT*: Wild-type
*Y1H*: Yeast one-hybrid
