# Supplementary material for: CmVNI2-CmMYB3 module regulates flavonol biosynthesis in response to low temperature in chrysanthemum flower
Source: Mol Hortic. 2025 Oct 9;5:53. doi: 10.1186/s43897-025-00175-x (PMC12509343; doi:10.1186/s43897-025-00175-x)
Supplement: Supplementary file 1 — Supplementary Material 1: Supplementary Figure S1. Sequence alignment of NAC proteins. Supplementary Figure S2. Phylogenetic relationships of CmVNI2 and NACs with known function. Supplementary Figure S3. Flavonoid-targeted metabolite profiling analysis in flowers of WT and CmVNI2 RNAi lines. Supplementary Figure S4. Results of Gene Ontology biological process term enrichment of downregulated DEGs. Supplementary Figure S5. Analysis of the CmVNI2 binding site length. Supplementary Figure S6. CmVNI2 influences the expression of SG19 MYBs and CmFLS. Supplementary Figure S7. Yeast one-hybrid analysis of CmVNI2 binding to the promoters of flavonol biosynthesis-related genes. Supplementary Figure S8. Flavonoid analysis of CmMYB3 overexpression in chrysanthemum using an UPLC-ESI–MS/MS system. Supplementary Figure S9. Flavonoid contents in CmMYB3 overexpression plants. Supplementary File S1. CmMYB3 promoter sequences. Supplementary Table S1. Number of total clean sequencing reads and mapping rate for each replicate of DAP-seq. Supplementary Table S2. Read count and fold enrichment in the fragment of the CmMYB3 promoter for each replicate of DAP-seq. Supplementary Table S3. List of primers used in this study. [file 43897_2025_175_MOESM1_ESM.docx]

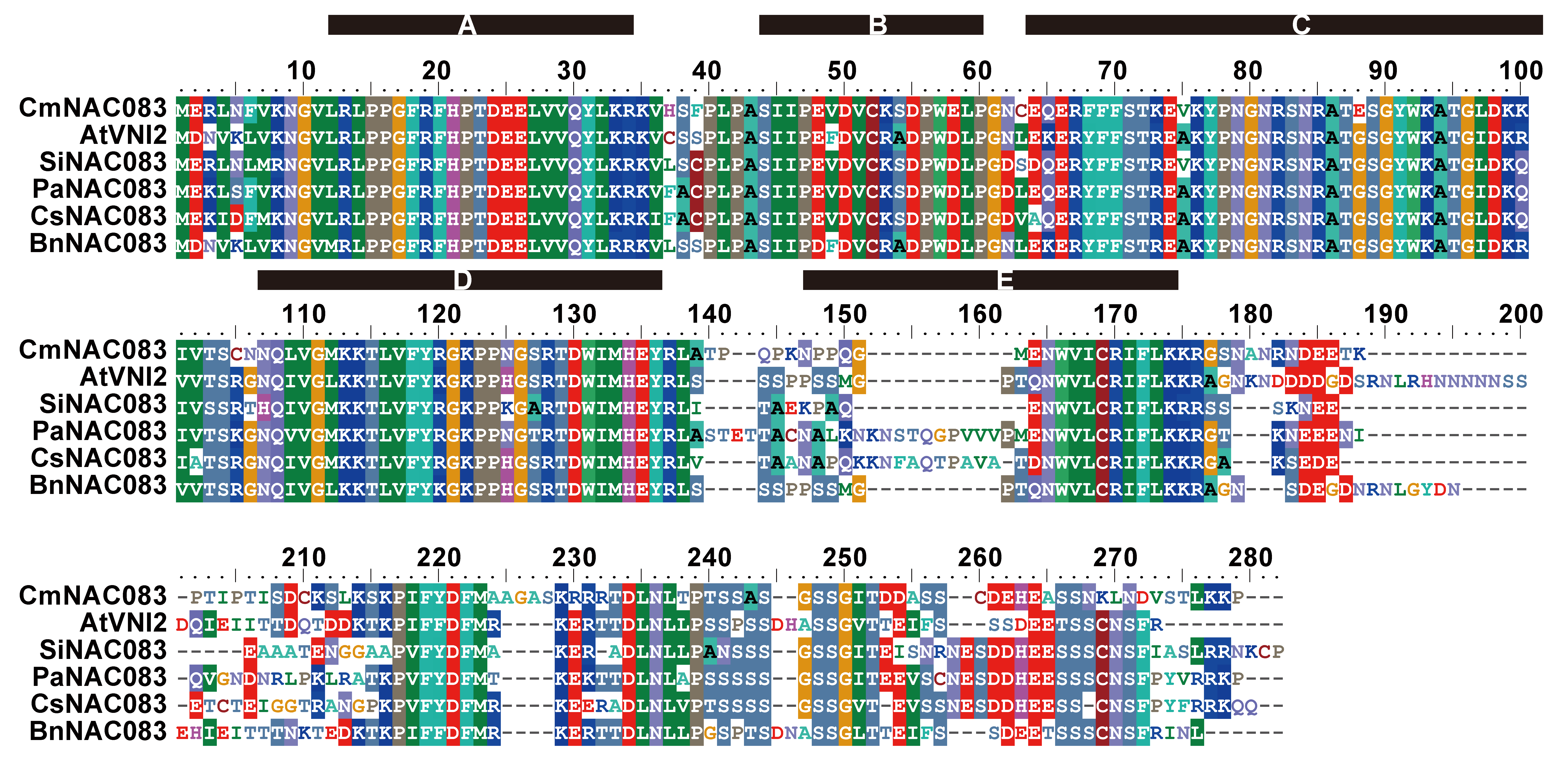


Figure S1 Sequence alignment of NAC proteins. Conserved NAC sub-domains are indicated by brown box. AtVNI2 (AT5G13180); SiNAC083 (XP_011086848.1); PaNAC083 (XP_034906860.1); CsNAC083 (KAH9774163.1); BnNAC083 (XP_013707659.1).


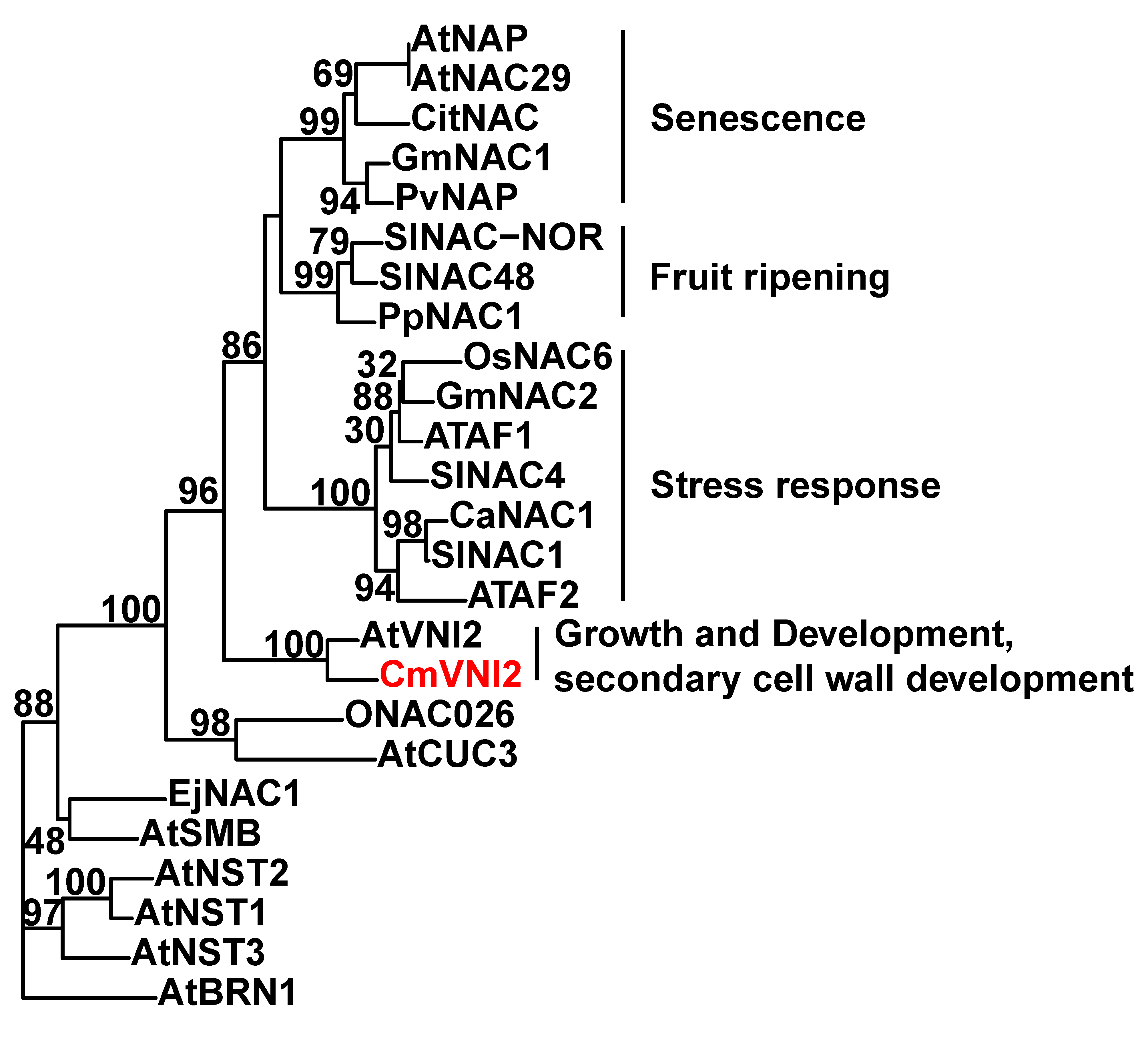


Figure S2 Phylogenetic relationships of CmVNI2 and NACs with known function. Numbers at the tree forks indicated bootstrap values. AtNAP (NM_105616.4); AtNAC29(NM_105616.4); CitNAC (ABM67699.1); GmNAC1 (NM_001249926.1); PvNAP (XM_007158582.1); SlNAC-NOR (AAU43923.2); SlNAC48 (AGT56012.1); PpNAC1 (ALK27820.1); OsNAC6 (BAA89800.1); GmNAC2 (NM_001249942.2); ATAF1 (ABD19694.1); SlNAC4 (AGH20611.1); CaNAC1 (AAW48094.1); SlNAC1 (NM_001247553.3); ATAF2 (BAC43493.1); AtVNI2 (AT5G13180); ONAC026 (APH07726.1); AtCUC3 (NM_106292.3); EjNAC1 (AJS13774.1); AtSMB (NM_001334893.1); AtNST2 (NM_116056.2); AtNST1 (NM_130243.3); AtNST3 (NM_103011.2); AtBRN1 (NM_103057.4).


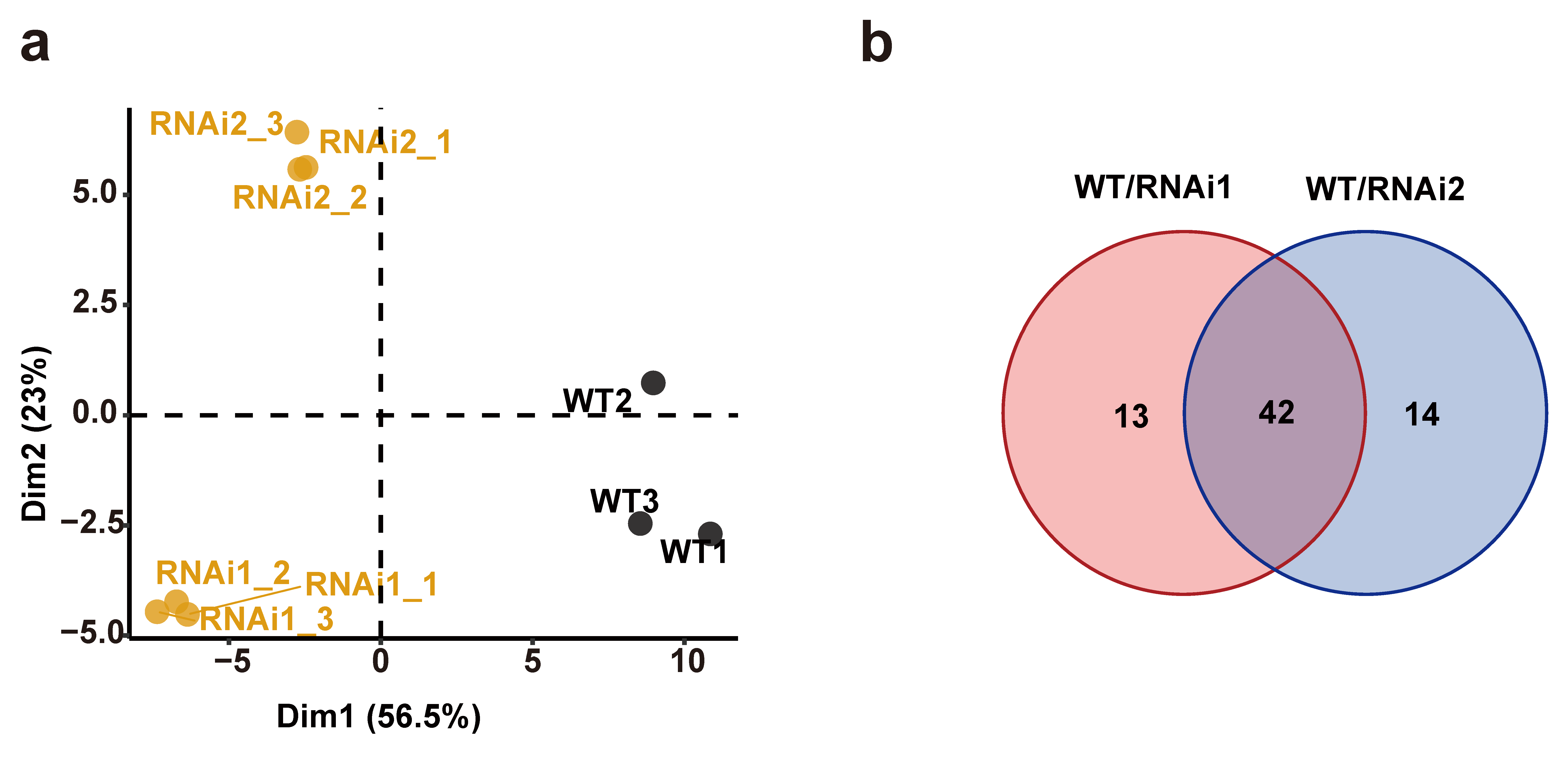


Figure S3 Flavonoid-targeted metabolite profiling analysis in flowers of WT and *CmVNI2* RNAi lines. a Principal component analysis (PCA) plot of flavonoid-targeted metabolic profiling dataset. The values in parentheses indicates the percentage of variance explained. b Interactions of differently accumulated flavonoid (DAF) in two *CmVNI2* RNAi lines compared to WT.


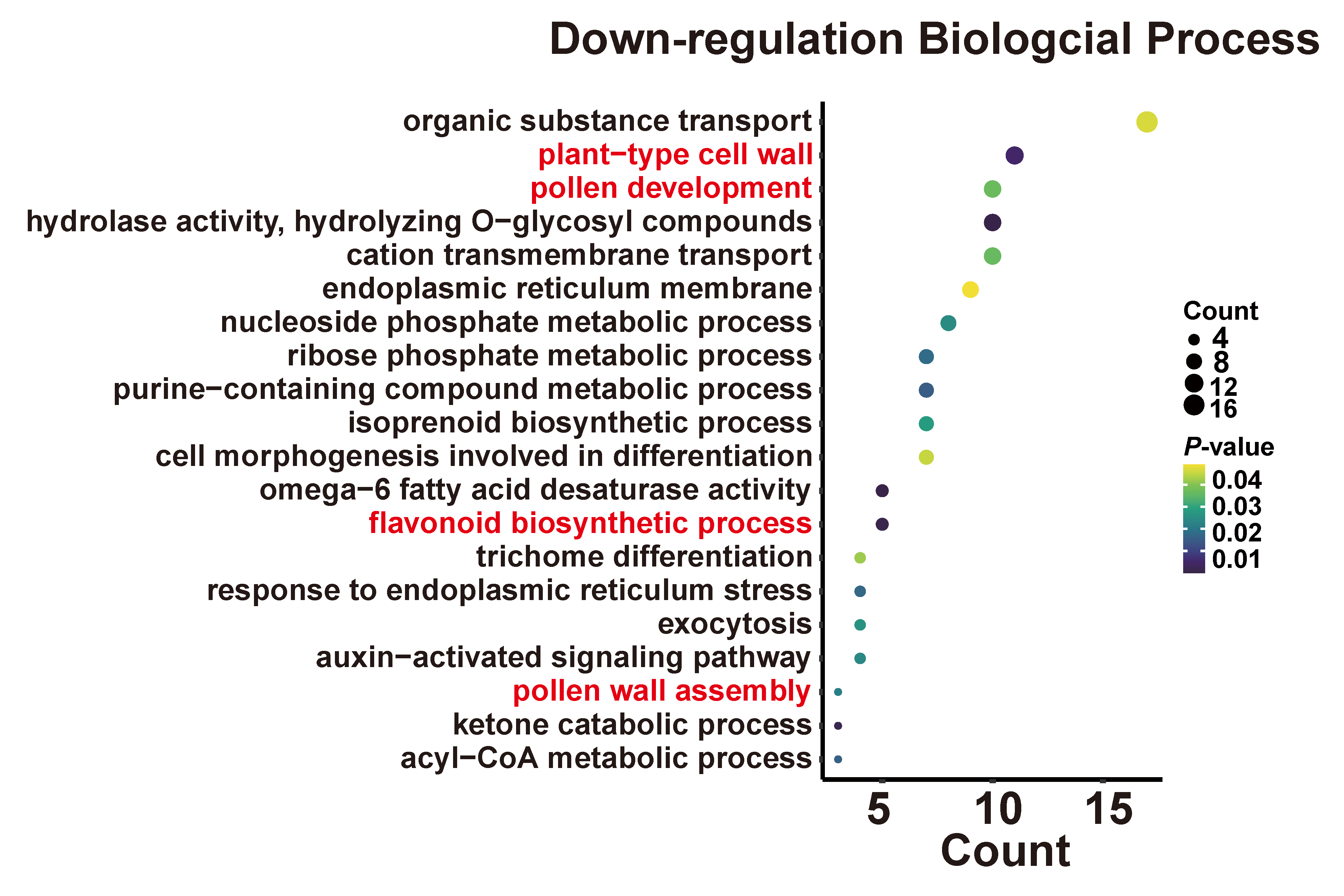


Figure S4 Results of Gene Ontology biological process term enrichment of downregulated DEGs.

| 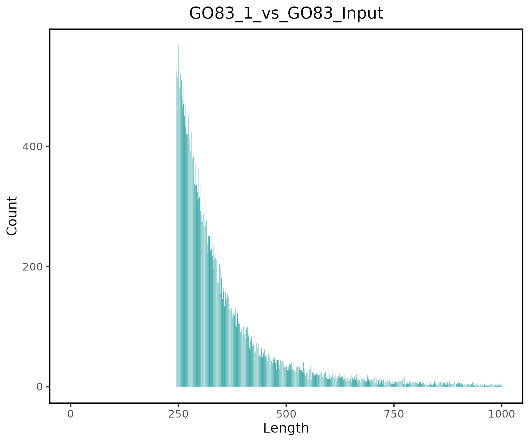 | 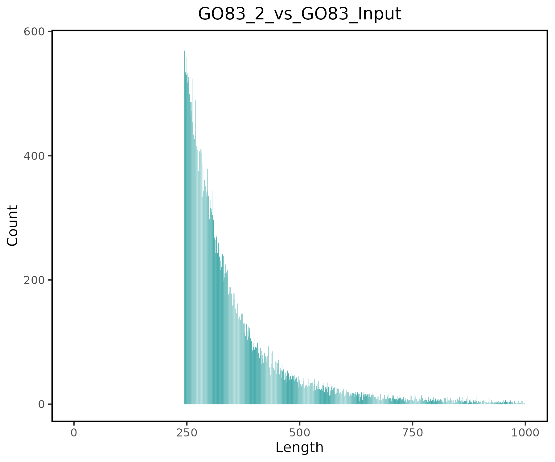 |
| --- | --- |

Figure S5. Analysis of the CmVNI2 binding site length. Histogram showing the length of the CmVNI2 binding peaks. Most binding peaks range from 250-1,000


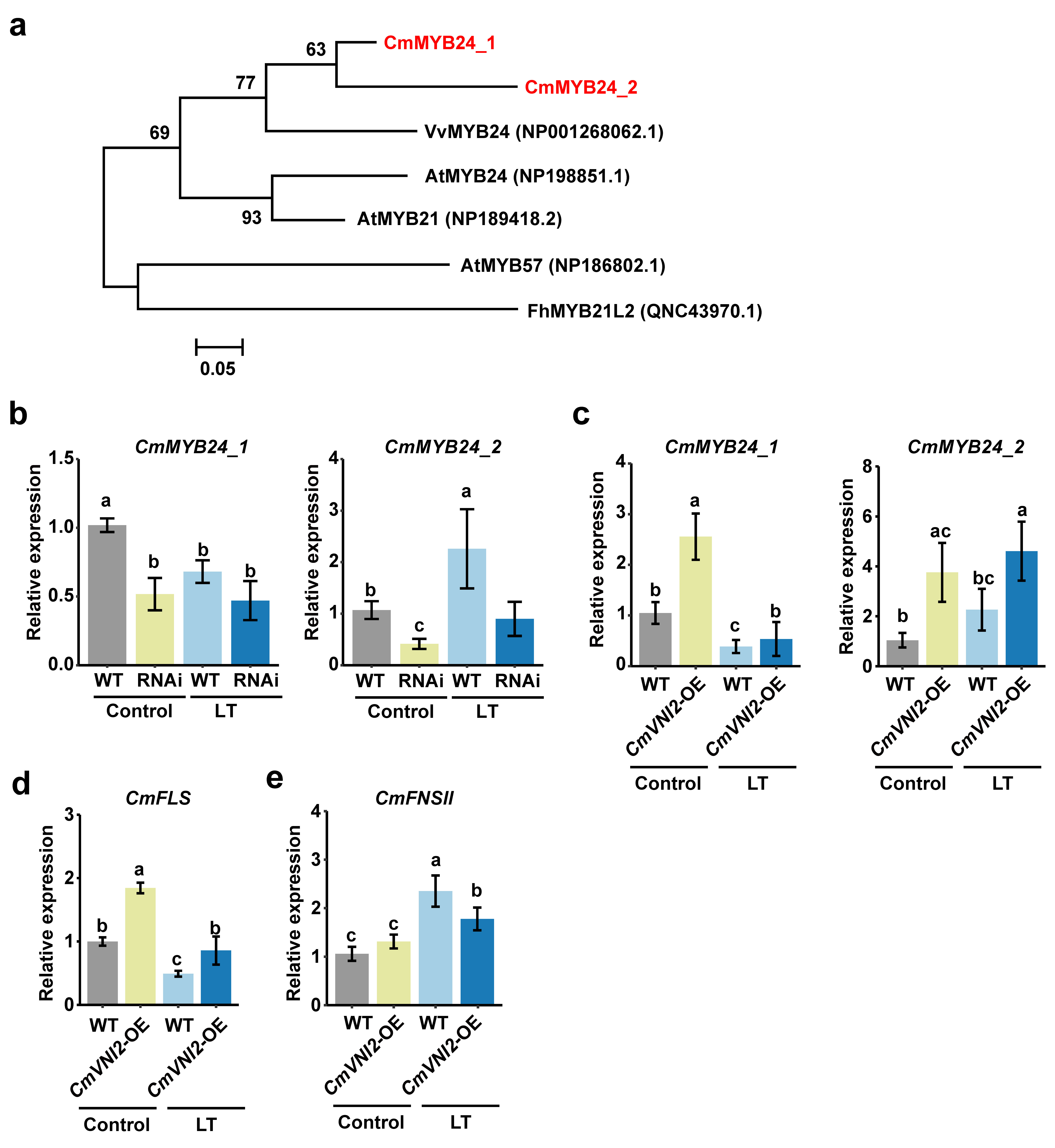


Figure S6. **CmVNI2 influences the expression of *SG19 MYB*s and *CmFLS*.** a Phylogenetic analysis of SG19 MYBs paralogs with other plant species. SG19 MYB family members in *C. indicum* are shown in red. The phylogenetic tree was constructed using the maximum likelihood method in MEGA (version 6). Bootstrap values from 1000 replicates for each branch are shown. b. Expression of SG19 MYB genes in WT and *CmVNI2* RNAi lines under control and low temperatures. Control, pre-treatment at 22°C; LT, following 7-day low temperature exposure at 10°C. c. Expression of SG19 MYB genes in WT and *CmVNI2*-OE flowers under control and low temperatures. d. Expression of *CmFLS* in WT and *CmVNI2*-OE flowers under control and low temperatures. e. Expression of *CmFLS* in WT and *CmVNI2*-OE flowers under control and low temperatures. The data are shown as the means ± SDs (n=3). Significantly different values (*P* < 0.05) are indicated with lowercase letters and were calculated via one-way ANOVA followed by Tukey’s HSD (honestly significant difference) test.


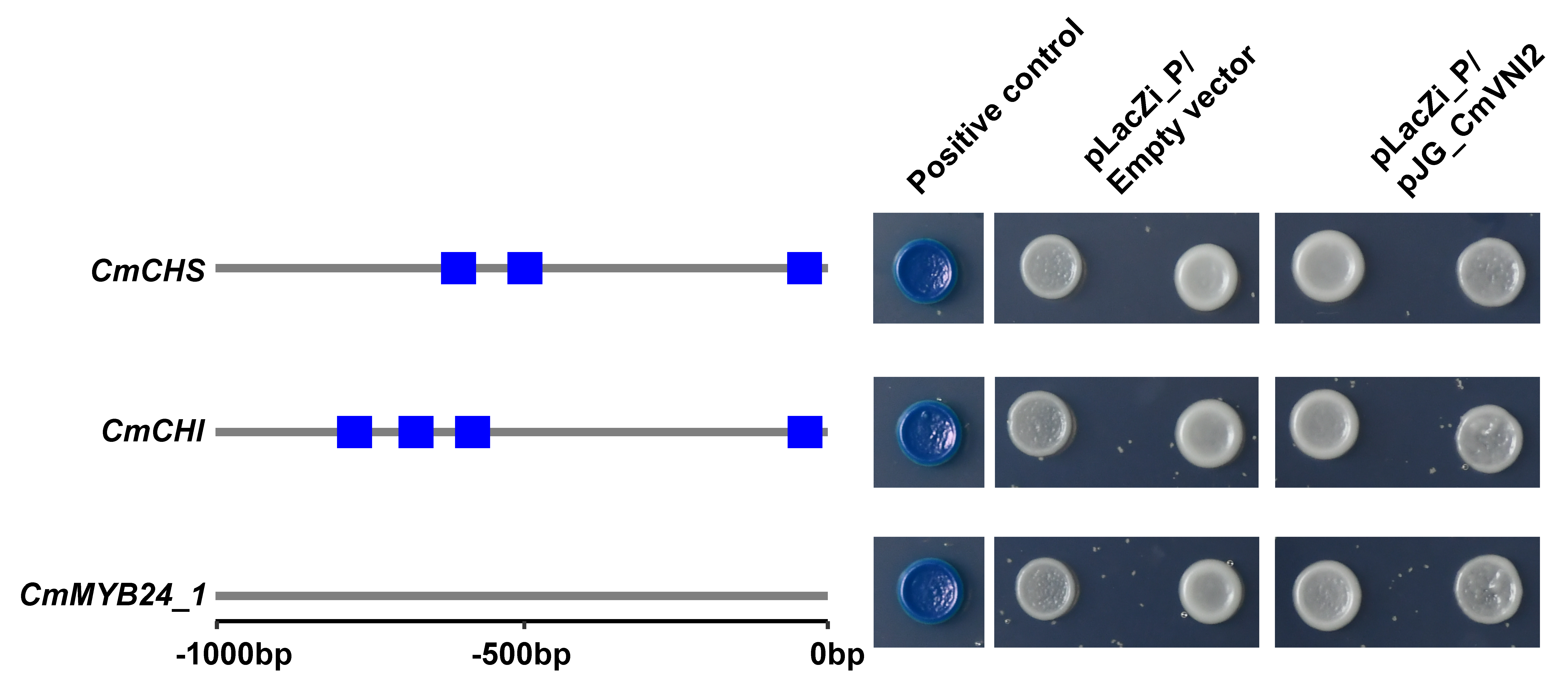


Figure S7 Yeast one-hybrid analysis of CmVNI2 binding to the promoters of flavonol biosynthesis related genes. Blue boxes within the promoter regions represent the potential NAC-binding cis-elements predicted using the JASPAP database (<https://jaspar.genereg.net/collection/core>). Interactions were determined by yeast cell growth and confirmed by colour indication on SD-Trp-Ura medium containing X-gal.


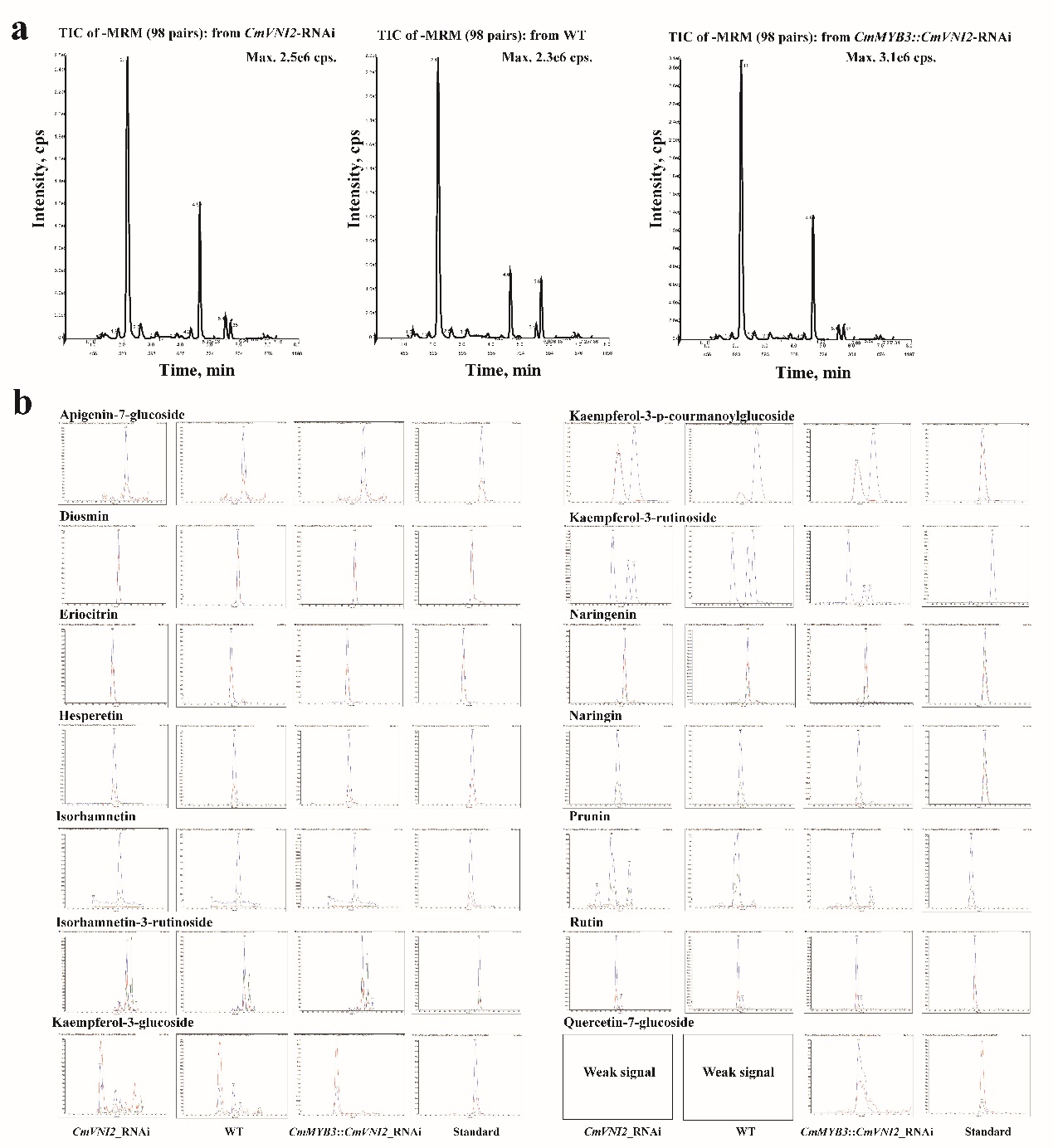


Figure S8 Flavonoid analysis of *CmMYB3* overexpression in chrysanthemum using an UPLC-ESI-MS/MS system. a Total ion chromatography (TIC) of flavonoids in leaves of *CmVNI2* RNAi, WT, and *CmMYB3+CmVNI2* RNAi plants. b Extracted ion chromatography (EIC) of flavonoids in leaves of *CmVNI2* RNAi, WT, and *CmMYB3+CmVNI2* RNAi plants.


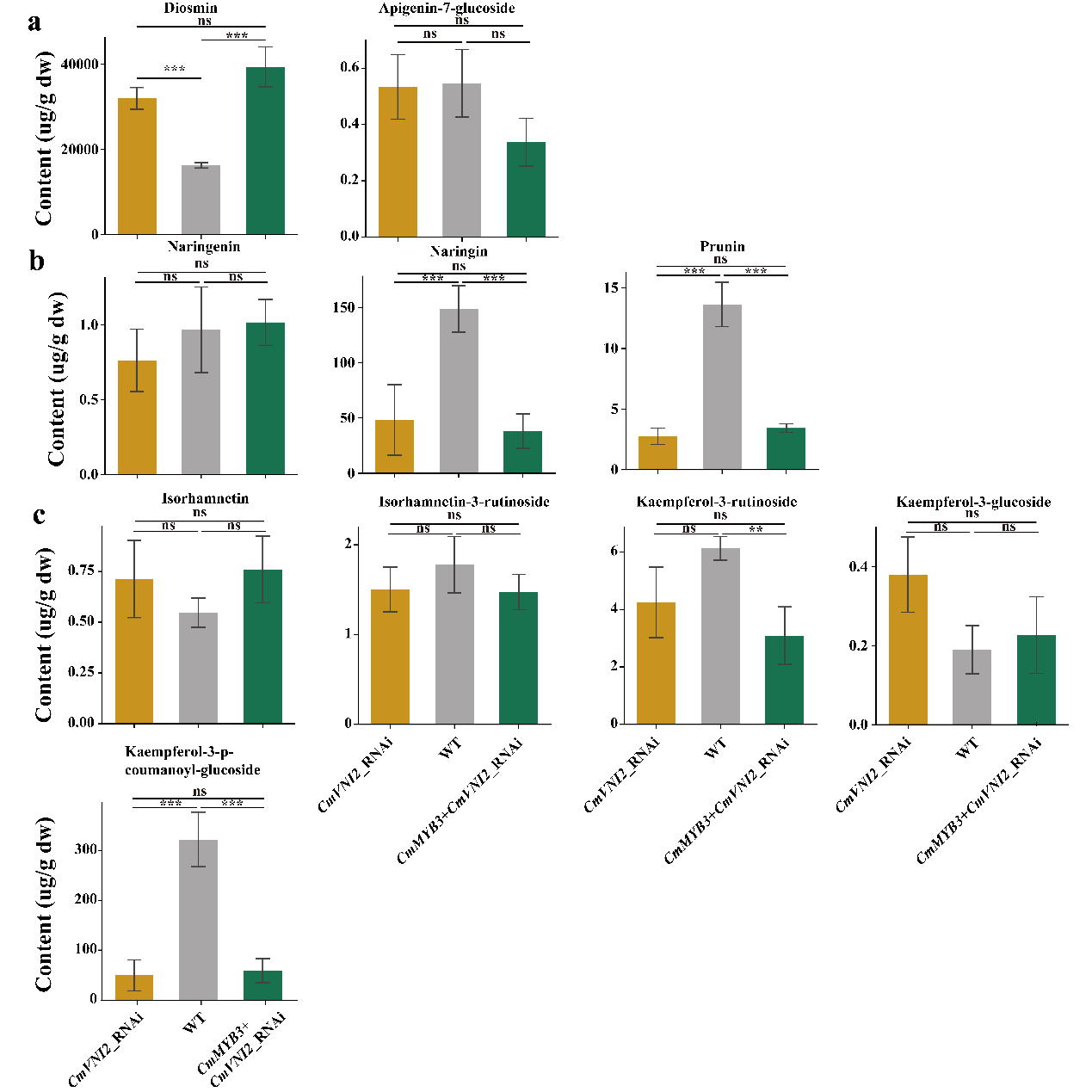


Figure S9. Flavonoid contents in *CmMYB3* overexpression plants. a, b, c Flavone, flavanone, and flavonol contents in leaves of WT, *CmVNI2* RNAi, and *CmMYB3* transient overexpression of *CmVNI2* RNAi plants (*CmMYB3*+*CmVNI2* RNAi). Empty vector (GFP) transformed leaves were used as controls. Values are means ± SD. Standard error bars were calculated from three biological replicates, sampled as described before. Asterisks indicate significant differences (Student’s *t*-test; **P* < 0.05; ***P* < 0.01; ****P* < 0.001).

**Supplemental File S1.** *CmMYB3* promoter sequences.

GTCTCTGATCACCCATAGTGATTTACTAATTATGCGATCCGTAATTTAAGGAAATCTTATAACAAAAGGGCATGATCAATACTAAGCTAGAAGGTATAGTAGTAGTAGGTTTTTGACAATGTCAATCTGGCCAATAATATCGATCACTCCCTTAATTCATAACAACAAACTCTATTATACTCAGTTAATGGTAGATAGTATGAAAATGTGAGGTAGATTCAAGAGTTGGTGAACACACCACTTAGAGATTATGTTTGTTACTCAATATCGTATTCTTGCGCGTTACATAGGCCAGTTACGTATACATGGGTCAAACAGTATGTGTTGGTTCAATTCAGCGCCGAGTAGTTTTTGAACATACTTTAACATAATAAGAATCTAAACATTGAAAAACTTAGTATATGTAAATCATGTTTAAAATTGAACGTAGTTGACACAAAACTGTTTTAAACTAAATTGTGTGTCTTTTGGAAAGTAATATATTTTCACCGTCGTTTTTTTTTATTTTTAAAAATAACTAAAATTTGTGTGCCACGAAAATTTGTTGTATAACTTAGGTTATACTTTGCAACCACTTTAAAAGGAATAACAGTGCGAGAAGTTTTTTGGTAAAGAATAAAGATATTACAGTACTAGTTAGGAGACTGACAGACCGTAACGAACGGCTAAATCAAAGTAACTATTTTACATTAGAAAAGAAAATGCACAAGTAAAGCTTCACACATATATTCTATGAAATAAATATGTAATTATGCATAACGTTAGGCACACCAATCATTTTTCAAGCCGTAAAGTACGGAGGCAAAGAAAAACGGTTGGATAGAATCAGACAAAACAATACAAAAAGTACCGACACAACTTTTGAACATTTGTTTTACGACAATGACTGTGGGTGAATGTTGTTCCACGGTACTAACGATGATTATCCGAGAGGATGATCAGTCACACTATGATCAAGACATGACCAAAACCAAAATTGAATTAGAAAAGCCCTAGAGAGAAATTGAAAGGGGGTTGAGAAAACGAGATAATATGGGGGGTGAAACGTGGGAGATTGATCTTCCGTTACTAGCCCTAGATACTAAAGATGCCACGACAAACTTAAAGTACTTGCTAATTTACGAGTAACATCATACCTCGTGGCAACAATTTCACCCACACACATACACACTTATTACATCATCACCCTCTTTTCATTTTCACTTTTTTTTTTTTTTTTTTTTTTTTTTTTTTTTTTTTTTTTTAATATACACACATTTATGTCCCTCAAACATCATACACTCTTTTAGTCTTACGTACATAATTGCTATAAATATCAAGCACTTACCCAAACCCCTATTGTACATCTTCTCATAAAATTTTGTCTCAAATATGGGAAGAGCACCATGTTGCCAAAAAATAGGGTTGAAGAGGGGAAGGTGGACCGCCGAAGAAGACAAGAT

Table S1 Number of total clean sequencing reads and mapping rate for each replicate of DAP-seq

| sample | totalReads | mapReads | uniqMapReads | rmDupReads |
| --- | --- | --- | --- | --- |
| GO83_1 | 72532096 | 71910344(99.14%) | 45172972(62.28%) | 42271612(58.28%) |
| GO83_2 | 73536032 | 72910890(99.15%) | 45695827(62.14%) | 42583619(57.91%) |
| GO83_Input | 66987900 | 66416892(99.15%) | 47106242(70.32%) | 44570263(66.53%) |

| sample | rawReads | rawBase | cleanReads | cleanBase | cleanQ20 | cleanQ30 | cleanGC |
| --- | --- | --- | --- | --- | --- | --- | --- |
| GO83_1 | 75561648 | 11.410G | 72532096(95.99%) | 10.855G(95.13%) | 98.83% | 96.19% | 37.97% |
| GO83_2 | 76601044 | 11.567G | 73536032(96.00%) | 11.008G(95.17%) | 98.87% | 96.31% | 37.89% |
| GO83_Input | 74901936 | 11.310G | 66987900(89.43%) | 10.041G(88.78%) | 98.98% | 96.62% | 42.09% |

Table S2 Read count and fold enrichment in the fragment of *CmMYB3* promoter for each replicate of DAP-seq

| **Sample** | **LG06:43832000-43834000_**  **reads count** | **Total reads count** | **Genome size** | **Reads scale** | **Fold enrichment** |
| --- | --- | --- | --- | --- | --- |
| GO83_1_vs_GO83_Input | 28 | 43048450 | 2.9×10^9^ | 0.943123388 | 1.973618073 |
| GO83_2_vs_GO83_Input | 34 | 43371472 | 2.9×10^9^ | 1.136691879 | 2.378687312 |
| GO83_Input | 15 | 45514928 | 2.9×10^9^ | 0.477865196 |  |

Table S3. List of primers used in this study

| **Primer Name** | **Forward (5’-3’)** | **Reverse (5’-3’)** |
| --- | --- | --- |
| *CmVNI2*_RNAi (*Asc* I-*Swa* I) | ttacaattaccatggggcgcgccTCTGATCCATGGGAGTTGCC | catgttcatctggggatttaaatCCAGTCGGTTCGTGATCCAT |
| *CmVNI2*_RNAi (*Bam H* I-*Pac* I) | cgatctctttgatggggatccCCAGTCGGTTCGTGATCCAT | gactctagggactagttaattaaTCTGATCCATGGGAGTTGCC |
| RT-qPCR-*CmVNI2* | TGATCCATGGGAGTTGCCAG | GTCGGTTCGCGATCCATTTG |
| RT-qPCR-*CmMYB3* | ACCGCCGAAGAAGACAAGAT | GCAACTCTTCCCACACCTCA |
| RT-qPCR-*CmF3H* | ACTACTCCAGGTGGCCTGAT | GCCTCTTTCTCAAGGCCCAT |
| RT-qPCR-*CmCHI* | TCCGATTGGGGTGCTAGAGA | TCCGGAATTCTTGAGGCGAC |
| RT-qPCR-*CmCHS* | CTCCTGAAAGACGTACCGGG | TCCTCCTTGAGACCGAGCTT |
| RT-qPCR-*CmFLS* | AACCAGCAACGACCACTCTC | ATACCCCAATCCTTGCTCGC |
| RT-qPCR-*CmFNSII* | TGCTGGCACCATGTTATTCG | CAAAGCACTGGATGAGACCG |
| RT-qPCR-MYB24_1 | ATCTCCGACCCGATGTAAGG | TTGCCCACTGAAGTTTTCCG |
| RT-qPCR-MYB24_2 | GAGTTGCATGCTAAGTGGGG | TAGTATTGTCGCACGGGTGA |
| RT-qPCR-*CmUBI3* | AGCTGAGCAGACTCCCGATG | AGGCGAATCATCAGTACCAAGT |
| *CmNAC*_ORF | gattatgcctctcccgaattcATGGAGAGGCTAAACTTTG | agaagtccaaagcttctcgagTTACGGTTTTTTCAAAGTGG |
| Y1H-pAbAi-*CmMYB3*-promoter | cttgaattcgagctcggtaccACGGAGGCAAAGAAAAACGG | atacagagcacatgcctcgagATCTTGTCTTCTTCGGCGGT |
| Y1H-pAbAi -*CmF3H*-promoter | cttgaattcgagctcggtaccCGGCCATCTGCACATATTC | atacagagcacatgcctcgagAAGTAGTTCACGAACTGCAGG |
| Y1H-pGADT7-*CmVNI2* | gtaccagattacgctcatatATGGAGAGGCTAAACTTTGTG | gattcatctgcagctcgagTTACGGTTTTTTCAAAGTGG |
| LUC-*CmMYB3*-promoter | gggtaccgggccccccctcgagGTCTCTGATCACCCATAGTG | gcaggaattcgatatcaagcttATCTTGTCTTCTTCGGCGGT |
| LUC-*CmF3H*-promoter | gggtaccgggccccccctcgagCGGCCATCTGCACATATTC | gcaggaattcgatatcaagcttCACGGACGAACCGGTTTTC |
| SK-CmVNI2 | atcccccgggctgcaggaattcATGGAGAGGCTAAACTTTG | tggtaccgggccccccctcgagTTACGGTTTTTTCAAAGTGG |
| EMSA-*CmF3H*-5’ Biotin probe | CCAACATTACGTACAAAACA | GGTTGTAATGCATGTTTTGT |
| EMSA-*CmF3H*-Competitor probe | CCAACATTACGTACAAAACA | GGTTGTAATGCATGTTTTGT |
| EMSA-*CmF3H*-Mutate probe | CCAACAGGGGGGGGGGAAAACA | GGTTGTAATGCATGTTTTGT |
| EMSA-*CmMYB3*-5’ Biotin probe | TTTTAGTCTTACGTACATAA | AAAATCAGAATGCATGTATT |
| EMSA-*CmMYB3*-Competitor_probe | TTTTAGTCTTACGTACATAA | AAAATCAGAATGCATGTATT |
| EMSA-*CmMYB3*-Mutate_probe | TTTTAGGGGGGGGGGGATAA | AAAATCCCCCCCCCCCTATT |
